# Dual VEGFA/BRAF targeting boosts PD‐1 blockade in melanoma through GM‐CSF‐mediated infiltration of M1 macrophages

**DOI:** 10.1002/1878-0261.13450

**Published:** 2023-05-27

**Authors:** Valentina Comunanza, Chiara Gigliotti, Simona Lamba, Gabriella Doronzo, Edoardo Vallariello, Valentina Martin, Claudio Isella, Enzo Medico, Alberto Bardelli, Dario Sangiolo, Federica Di Nicolantonio, Federico Bussolino

**Affiliations:** ^1^ Department of Oncology University of Torino Candiolo Italy; ^2^ Candiolo Cancer Institute FPO – IRCCS Candiolo Italy; ^3^ IFOM ETS The AIRC Institute of Molecular Oncology Milan Italy

**Keywords:** BRAF, M1‐macrophage, melanoma, VEGFA

## Abstract

The introduction of targeted therapies represented one of the most significant advances in the treatment of BRAFV600E melanoma. However, the onset of acquired resistance remains a challenge. Previously, we showed in mouse xenografts that vascular endothelial growth factor (VEGFA) removal enhanced the antitumor effect of BRAF inhibition through the recruitment of M1 macrophages. In this work, we explored the strategy of VEGFA/BRAF inhibition in immunocompetent melanoma murine models. In BRAF mutant D4M melanoma tumors, VEGFA/BRAF targeting reshaped the tumor microenvironment, largely by stimulating infiltration of M1 macrophages and CD8^+^ T cells, and sensitized tumors to immune checkpoint blockade (ICB). Furthermore, we reported that the association of VEGFA/BRAF targeting with anti‐PD‐1 antibody (triple therapy) resulted in a durable response and enabled complete tumor eradication in 50% of the mice, establishing immunological memory. Neutralization and CRISPR‐Cas‐mediated editing of granulocyte‐macrophage colony‐stimulating factor (GM‐CSF) abrogated antitumor response prompted by triple therapy and identified GM‐CSF as the cytokine instrumental in M1‐macrophage recruitment. Our data suggest that VEGFA/BRAF targeting in melanoma induces the activation of innate and adaptive immunity and prepares tumors for ICB. Our study contributes to understanding the tumor biology of BRAFV600E melanoma and suggests VEGFA as therapeutic target.

## Graphical abstract

The authors report that BRAF/VEGFA targeting reverses immune cell exclusion by increasing the infiltration of CD8 T cells and M1–macrophages, allowing remodeling of the tumor microenvironment and sensitizing melanoma cells to immune checkpoint blockade. This offers potential for a new treatment strategy based on targeting VEGFA that aims to overcome resistance to BRAF blockade in melanoma.

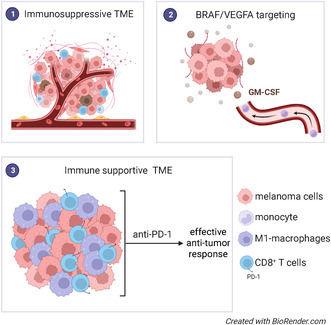

**Abbreviations**
BRAFi: BRAF inhibitor
CR: complete response
CRISPR: clustered regularly interspaced short palindromic repeats
CSFR1: colony‐stimulating factor‐1 receptor
DR: durable response
GM‐CSF: granulocyte‐macrophages colony‐stimulating factor
HIF‐1‐α: hypoxia‐inducible factor 1‐α
hVEGFA: human vascular endothelial growth factor A
ICB: immune checkpoint blockade
MDSCs: myeloid‐derived suppressor cells
MEKi: MEK inhibitors
MHCII: major histocompatibility complex II
M‐MDSC: monocytic myeloid‐derived suppressor cell
mVEGFA: murine vascular endothelial growth factor A
PD‐1: programmed cell death protein 1
PD‐L1: programmed death‐ligand 1
PMN‐MDSC: polymorphonuclear myeloid‐derived suppressor cell
qRT‐PCR: quantitative reverse transcription‐polymerase chain reaction
SR: short response
TAMs: tumor‐associated macrophages
TME: tumor microenvironment
VEGFA: vascular endothelial growth factor A

## 1 Introduction

Targeted therapies, such as BRAF (BRAFi) and MEK (MEKi) inhibitors, represent one of the most significant advances in the treatment of BRAFV600E melanomas. However, despite the progress in the management of combinatorial therapies, the development of acquired resistance represents an urgent clinical need. At the same time, immune checkpoint blockade (ICB) is a promising approach independently from the melanoma mutational landscape and provide potential for durable response. Nevertheless, a fraction of melanoma patients fails to respond to these interventions [1]. It has been demonstrated that the composition of tumor microenvironment (TME) may influence response to ICB. For this reason, strategies aimed to modulate the TME to sensitize refractories tumors are under investigations to enhance the response to ICB [2].

Targeting oncogenic BRAF makes melanoma more immunogenic and promotes a more favorable TME by normalizing tumor vasculature, enhancing melanoma antigen presentation, and inducing cytotoxic CD8^+^ cells infiltration [3, 4, 5, 6]. In BRAF mutant melanomas, the combination of targeted and immunotherapy has been tested in preclinical models [7], and clinical trials are currently underway [8, 9], suggesting that a combination of BRAFi, MEK inhibitor, and PD‐1 blockade may be feasible. However, additional options are needed for specific subsets of patients.

Vascular endothelial growth factor A (VEGFA) is a potent angiogenic factor involved in tumor progression. On the contrary, VEGFA is critical for promoting immune suppressive activity, impairing leukocyte diapedesis and hinders the immune T effector cell infiltration into the tumors [10]. Also, VEGFA hampers tumor T‐cell development [11] and correlates with PD‐1 expression of CD8^+^ cells [12]. In addition to direct effects on T cells, VEGFA suppresses dendritic cell functions and expands T regulatory cells and myeloid‐derived suppressor cells (MDSCs) [13, 14, 15].

Interestingly, VEGFA blockade has a positive impact on the immune mechanism leading to the antitumor response [16] and preclinical and clinical studies support the possibility to exploit angiogenesis inhibitors in association with immunotherapy [17, 18, 19].

In colorectal cancer, bevacizumab improved the antigen‐presenting capacity of dendritic cells [20] revealing an additional mechanism for VEGFA‐removal on immune functions in the context of ICB. Furthermore, high serum levels of VEGFA were associated with decreased overall survival in advanced melanomas [21], while low plasmatic VEGFA characterized patients responding to immunotherapy [22]. Finally, the addition of bevacizumab to anti‐PD‐L1 antibody plus standard chemotherapy improved overall survival among patients with metastatic non‐small‐cell lung carcinoma [23].

Previously, we reported that bevacizumab, the anti‐human VEGFA monoclonal antibody (anti‐hVEGFA), delayed the onset of acquired resistance to BRAFi in a human melanoma tumor model developed in immunodeficient mice. Such anticancer effect was related to the recruitment of M1‐like macrophages and largely limited by macrophage depletion [24].

Here, we sought to test the hypothesis that the antitumor efficacy induced by the combinatorial use of BRAFi and VEGFA removal, BRAF/VEGFA targeting, correlates with the ability to induce an immune‐stimulatory milieu in a mouse syngeneic melanoma tumor model, derived from BRAF mutated murine cells. We identified that BRAF/VEGFA targeting induced a granulocyte‐macrophages colony‐stimulating factor (GM‐CSF)–mediated recruitment of M1 macrophages. Combinatorial BRAFi and VEGFA removal reshapes the TME maximizing the anticancer effects of ICB therapies, suggesting that VEGFA neutralization represents a further option to improve targeted therapy and immunotherapy in melanoma.

## 2 Materials and methods

### 2.1 Cell lines

BRAF mutant D4M (D4M.7A; RRID:CVCL_0P29) mouse melanoma cells were generated from Tyr::CreER;BrafCA;Ptenlox/lox mice [25] and were purchased by Kerafast (Boston, MA, USA). BRAF mutant 5555 mouse melanoma cells were established from C57BL/6_BRAF+/LSL‐BRAFV600E;Tyr::CreERT2+/o [26] and were kindly provided by R. Marais (Cancer Research UK Manchester Institute). D4M cells were cultured in DMEM/F‐12 advanced media (Sigma‐Aldrich, St. Louis, MO, USA) and supplemented with 5% FBS, 1% of penicillin/streptomycin (Sigma‐Aldrich), and 2 mm glutamine (Sigma‐Aldrich). 5555 cells were cultured in DMEM (Sigma‐Aldrich) and supplemented with 10% FBS, 1% of penicillin/streptomycin (Sigma‐Aldrich), and 2 mm glutamine (Sigma‐Aldrich). The cells were grown according to standard protocols in a 37 °C humidified, 5% CO_2_ incubator, and were tested regularly for mycoplasma contamination. To authenticate cell lines, cells were confirmed for BRAFV600E mutation by PCR analyses and *in vitro* sensitivity to PLX4720.

### 2.2 Animal studies

C57BL/6 were purchased from Charles River (Calco, Como, Italy) and were acclimatized in the animal colony for 1 week before experimentation. D4M (5 × 10^5^) and 5555 (2 × 10^6^) cells were resuspended in PBS and Matrigel and subcutaneously injected in 6–8‐week‐old C57BL/6 female mice. Tumor size was measured with a caliper, and tumor volume was calculated by the modified ellipsoid formula: [length × (width)^2^/2]. When tumors reached a volume of approximately 250 mm^3^, mice were randomly assigned to different treatment groups, which were maintained for 12 days or 8 weeks. Animals were housed in a sterile environment, individually ventilated cages containing autoclaved bedding, food, and water. Animals were kept under supervision by veterinary personnel during all the experiments throughout the entire duration of the experiments. Mice animals were not previously involved in other experimental procedures and were monitored for social behaviors, compromised motility, and sign of distress. The investigators did not operate in blind. All animal procedures were approved by Italian Ministry of Health (protocol 21635.13) and were performed in accordance with institutional guidelines and international law and policies.

### 2.3 Mouse treatments

BRAFi (PLX4720) was purchased from Selleck Chemicals (Houston, TX, USA), dissolved in DMSO at a final concentration of 500 mm and stored in aliquots at 80 °C. PLX4720 was administered by daily oral gavage at the dosage of 60 mg·kg^−1^ (dissolved in a vehicle of 1% w/v methylcellulose in sterile water). The anti‐murine VEGFA (anti‐mVEGFA; B20) was provided by Genentech Inc. (San Francisco, CA, USA) and administered intraperitoneally three times a week (10 mg·kg^−1^, diluted with sterile 0.9% saline). The anti‐mouse PD‐1 (clone RMP1‐14), anti‐mouse CD8a (clone YTS169.4), anti‐GMCSF (clone MP1‐22E), anti‐CSFR1 (clone AFS98), rat IgG2a (clone 2A3), and rat IgG2b (clone LTF‐2) were purchased from BioXcell (Lebanon, NH, USA). Antibodies were diluted with sterile 0.9% saline Anti‐PD‐1 was administered intraperitoneally three times a week (250 μg per mouse). Anti‐mouse CD8a was used for depletion of CD8^+^ T cells in immunocompetent mice and was injected intraperitoneally on the day of tumor inoculation (400 μg per mouse) and every 3 days throughout the course of the experiments (200 μg per mouse). Anti‐mouse, GM‐CSF, was injected intraperitoneally on the day of tumor inoculation and every 3 days throughout the course of the experiments (250 μg per mouse). Anti‐mouse CSFR1 was injected intraperitoneally on the day of tumor inoculation and every 3 days throughout the course of the experiments (500 μg per mouse). Isotype controls were injected according to the same schedule.

### 2.4 Gene expression data

Microarray gene expression data set were downloaded from Gene Expression Omnibus dataset (GEO; https://www.ncbi.nlm.nih.gov/geo/; accession number GSE69754). Probes characterized by at least one experimental condition with *P* < 0.05 were selected. Cross species hybridizing probes were retrieved from [27] and filtered out. For each gene, we retained the associated probe with the largest mean expression value across all samples. Ligand receptor database was generated as comprehensive compendium of interactions by assembling two resources: IUPAR (https://www.guidetopharmacology.org) and ligand–receptor databases (https://dip.doe‐mbi.ucla.edu/dip/DLRP.cgi) for a total of 356 interactions.

### 2.5 Immunofluorescence analysis

Immunofluorescence was performed on frozen and fixed tumor sections. The antibodies used were: anti‐mouse F4/80 (MCA497G; Bio‐Rad Laboratories, Hercules, CA, USA), GM‐CSF (ab9741; Abcam, Cambridge, UK), CD68 (ab125212; Abcam), phosho‐p44/42 MAPK (erk1/2) (4370; Cell Signaling Technology, Danvers, MA, USA), and were revealed with the appropriate fluorescence‐conjugated secondary antibodies (Alexa 647 or 488). Images were analyzed by Leica SPEII confocal microscope (Leica microsystem, Wetzlar, Germany). Multiple independent fields (15–20 for section) were randomly chosen and analyzed from at least three tumors for each experimental condition. Image quantification was performed using ImageJ software (National Institutes of Health, Bethesda, MD, USA).

### 2.6 Quantitative real‐time RT‐PCR

Gene expression analysis by real‐time quantitative RT‐PCR (qRT‐PCR) were performed on F4/80^+^ cells magnetically sorted *ex vivo* with anti‐F480 MicroBeads Ultrapure (Miltenyi Biotec, Bergisch Gladbach, Germany). The following primers were used: CCl5, forward 5′‐GACAGCACATGCATCTCCCA‐3′ and reverse 5′‐GTGTCCGAGCCATATGGTGA‐3′; Cd40, forward 5′‐TTGTTGACAGCGGTCCATCT‐3′ and reverse 5′‐TCTCAAGAGCTGTGCAGTGG‐3′; Cd86, forward 5′‐CAGCACGGACTTGAACAACC‐3′ and reverse 5′‐CTCCACGGAAACAGCATCTGA‐3′; Cxcl10, forward 5′‐GAGAGACATCCCGAGCCAAC‐3′ and reverse 5′‐GGGATCCCTTGAGTCCCAC‐3; Cxcl9, forward 5′‐TGGAGTTCGAGGAACCCTAGT‐3′ and reverse 5′‐TTGTAGTGGATCGTGCCTCG‐3′; Tbp, forward 5′‐AGTGCCCAGCATCACTATTTCA‐3′ and reverse 5′‐GCCCTGAGCATAAGGTGGAA‐3′; Tnfa, forward 5′‐ GTAGCCCACGTCGTAGCAAA‐3′ and reverse 5′‐ ACAAGGTACAACCCATCGGC‐3′; Nos2, forward 5′‐CCTTGGTGAAGGGACTGAGC‐3′ and reverse 5′‐ CAACGTTCTCCGTTCTCTTGCT‐3′. Total RNA was extracted from tumors using Maxwell® RSC miRNA Tissue kit (AS1460; Promega, Madison, WI, USA). Reverse transcription (RT) and qRT‐PCR were performed as previously described [28] For cDNA synthesis, a High‐Capacity cDNA Reverse Transcription kit (Life Technologies, Carlsbad, CA, USA) was used according to the manufacturer's instructions. An RNA quality check, including concentration and purity, was performed with a Nanodrop ND‐100 spectrophotometer Thermo Fisher Scientific, Waltham, MA, USA). qRT‐PCR was performed on a CFX96 (Bio‐Rad Laboratories) using SYBR‐green PCR MasterMix (Life Technologies). The PCR thermal profiles were 95 °C for 15 s and 60 °C for 60 s (40 cycles). Melting curve analysis was performed for each PCR to confirm the specificity of the amplifications. The housekeeping gene TBP was used to normalize the expression data. Analyses of sorted cells from tumor‐bearing mice were performed using independent experiments from four mouse replicate for each treatment condition.

### 2.7 Tumor dissociation

Tumors were cut into small fragments (around 1 mm^3^) and transferred to a tissue digestion C‐tube (Miltenyi Biotec) and dissociated enzymatically and mechanically using a tumor dissociation kit (mouse; Miltenyi Biotec) on a gentleMACS. Dissociator (Miltenyi Biotec). Briefly, the “m‐impTumor‐02” program was run on the dissociator, followed by a 40‐min incubation at 37 °C.After a final run of the “m‐impTumor‐03,” a single‐cell suspension was obtained by filtering through a 70‐μm cell strainer (BD Biosciences, Franklin Lakes, NJ, USA). Single‐cell suspensions were centrifuge for 5 min at 300 ***g*** and washed with PBS.

### 2.8 Flow cytometry

Phenotype analysis was performed with staining performed at 4 °C for 20 min with the following antibodies: anti‐mouse CD45 (clone 30‐F11; Biolegend, San Diego, CA, USA), anti‐mouse F4/80 (clone BM8; Biolegend), anti‐mouse CD11c (clone N418; Biolegend), anti‐mouse Ly6C (clone HK1.4; Thermo Fisher Scientific); anti‐CD11b (clone M1/70; Biolegend, San Diego, CA, USA), anti‐CD206 (clone C068C2; Biolegend), anti‐Ly6G (clone 1A8; Biolegend), anti‐NK1.1 (clone PK136; Biolegend), anti‐CD314 (clone C7; Biolegend), anti‐CD4 (clone GK1.5; BD Pharmigen, San Jose, CA, USA), anti CD8 (clone 53‐6.7; BD Pharmigen); antiPD‐1 (clone 29F.1A12; Biolegend), anti‐PD‐L1 (clone 10F.9G2; Biolegend). Cells were detected using the Cyan ADP flow cytometer (Beckman Coulter, Brea, CA, USA) and data were analyzed with the Summit 4.3 software (Beckman Coulter). Quadrants were set based on isotype control antibody, and cells were gated among total DAPI^−^ cells.

### 2.9 Gene editing

The knockout of the *Csf2* gene in mouse cells was generated using the genome editing one vector system (lentiCRISPR‐v2) (Addgene #52961; Watertown, MA, USA). sgRNAs were designed using the CRISPR tool (http://crispr.mit.edu) to minimize potential off‐target effects. The following sgRNA sequences were used: sgRNA4: CCCTCACTCACCAACGTGAC; sgRNA6: GGCTGTAGACCACAATGCCC. Annealed sgRNA oligonucleotides targeting mouse *Csf2* were cloned into Bsmbl lentiCRISPR‐v2 plasmid, as previously described [25]. To express the CRISPR–Cas9 system transiently, we transfected cells with lentiCRISPR‐v2 vector plasmid (using the same guides as described above). Transfection was carried out using Lipofectamine 3000 (Life Technologies) and Opti‐MEM (Invitrogen), according to the manufacturer's instructions. After 48 h, cells were incubated with puromycin (Sigma‐Aldrich) for 4 days and subsequently single‐cell‐diluted in 96‐well plates. Approximately 40 clones for each guide were tested for the efficiency of editing. We selected clones that lacked *Csf2* and confirmed the absence of Cas9 on the basis of immunofluorescence analysis.

### 2.10 Statistics

All statistical analyses were performed using the graphpad prism 8.0 software (GraphPad Software, San Diego, CA, USA). All experiments were conducted with at least three replicates. To calculate statistical significance, two‐tailed Student's *t*‐test or one‐way analysis of variance (ANOVA) followed by Tukey's multiple comparisons test was used to determine the significance of differences between the indicated groups. Kaplan–Meier survival curves were compared between different treatment groups using the log‐rank (Mantel–Cox) test. Data were expressed as mean ± SEM. *P < 0.05, **P < 0.01, and ***P < 0.001 were regarded as statistically significant.

## 3 Results

### 3.1 Transcriptome analysis of A375 melanoma tumors treated with BRAFi and anti‐hVEGFA dual therapy unveils the upregulation of M1‐macrophage chemoattractant GM‐CSF

**Fig. 1:**
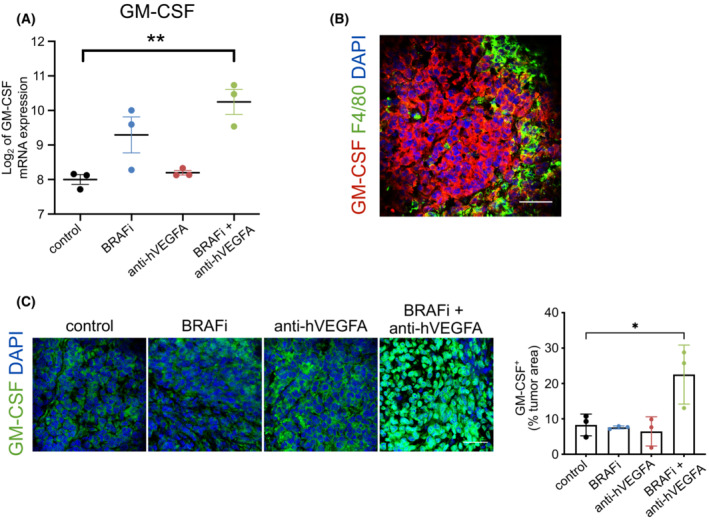
Ligand–receptor genes differentially expressed after treatment with BRAF inhibitor (BRAFi) associated with antihuman VEGFA antibody (anti‐hVEGFA) in A375 melanoma xenograft. (A) Log_2_‐transformed, normalized expression value of granulocyte‐macrophages colony‐stimulating factor (GM‐CSF) quantified by microarray in control, BRAFi, anti‐hVEGFA and BRAFi + anti‐h‐VEGFA A375 tumors. GM‐CSF ligand is detected in human dataset; *n* = 3. (B) Immunofluorescence staining for GM‐CSF (red) and F4/80 (green) protein in A375 xenograft treated with BRAFi in combination with anti‐hVEGFA. (C) Quantification of GM‐CSF expression determined by immunofluorescence staining in A375 tumors treated as indicated. Bar graphs indicated the GM‐CSF^+^ area/tumor area; *n* = 3. Scale bar, 40 μm. Data are presented as means ± SEM. Significance was assessed by and Student's *t*‐test (A) and one‐way ANOVA test followed *post hoc* pairwise analysis test (C); *P < 0.05, **P < 0.01.

We previously reported that BRAFi in association with anti‐hVEGFA bevacizumab promotes the infiltration of M1‐like macrophages, which show antitumor activity in melanoma A375 xenograft model [24]. In this study, we exploited the previous generated transcriptomes of A375 xenografts treated with BRAFi, bevacizumab, and their combination [24] focusing on the comparative analysis of differentially expressed human and murine mRNAs of ligand–receptor pairs to identify intercellular signals between cancer and stromal cells accountable for the observed modifications of TME after the therapeutic treatment. To highlight the potential paracrine mechanism of interaction between tumor cells and murine macrophages, we focused on ligands expressed by melanoma cells for which a cognate receptor was found expressed on stromal cells (for a total of 80 ligands and 72 receptors). We found five ligands differentially expressed only after dual therapy and, among these, the most upregulated ligand was GM‐CSF (*CSF2*) (Fig. 1A and Table S1). Its cognate receptors, Csfr2a expression on stroma cells, were not modulated by any of the treatments (Table S2). Interestingly, GM‐CSF expressing cells were evident in close proximity to tumor‐infiltrating F4/80^+^macrophages (Fig. 1B) and, in agreement with gene expression data, GM‐CSF protein significantly increased only after dual therapy (Fig. 1C).

### 3.2 VEGFA blockade and BRAFi are synergic and induces antitumor M1‐macrophage infiltration in D4M melanoma models

**Fig. 2:**
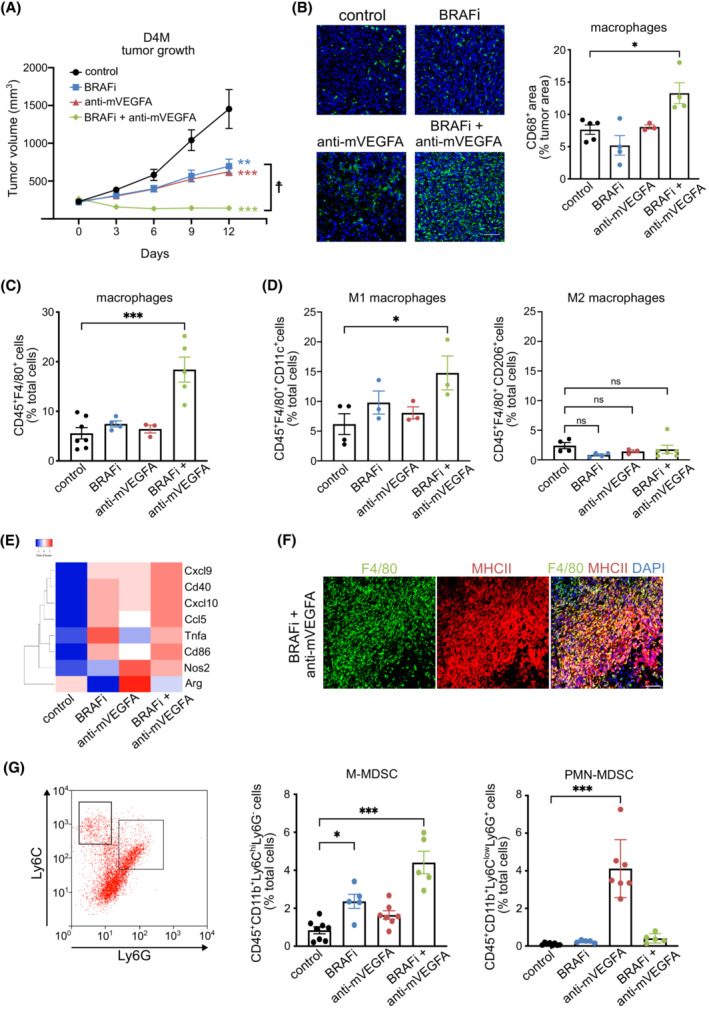
Vascular endothelial growth factor (VEGFA) targeting results in synergistic activity with BRAF inhibitor (BRAFi) in D4M syngeneic melanoma model. (A) Mice bearing established D4M tumors were treated for 12 days with control (*n* = 12), BRAFi (PLX470; *n* = 13), anti‐murine VEGFA antibody (anti‐mVEGFA, B20; *n* = 13) or BRAFi + anti‐mVEGFA (*n* = 14). (B) Representative images and quantification of macrophage infiltration determined by CD68 immunofluorescence staining in D4M melanoma tumors treated as indicated. Bar graphs indicated the CD68^+^ area/tumor area, (control *n* = 5, BRAFi *n* = 4, anti‐mVEGFA *n* = 3, BRAFi + anti‐mVEGFA *n* = 4). Scale bar, 50 μm. (C) Flow cytometry quantification of tumor infiltrating CD45^+^F4/80^+^ macrophages (control *n* = 7, BRAFi *n* = 4, anti‐mVEGFA *n* = 3, BRAFi + anti‐mVEGFA *n* = 5). (D) Flow cytometry quantification of tumor infiltrating CD45^+^F4/80^+^CD11c^+^ M1 macrophages (control *n* = 4, BRAFi *n* = 3, anti‐mVEGFA *n* = 3, BRAFi + anti‐mVEGFA *n* = 3) and CD45^+^F4/80^+^CD206^+^ M2 macrophages (control *n* = 4, BRAFi *n* = 4, anti‐mVEGFA *n* = 3, BRAFi + anti‐mVEGFA *n* = 6). (E) Heatmap representation of Log_2_ fold change of markers associated to M1 and M2 polarization phenotype detected by qRT‐PCR in in isolated macrophages D4M melanoma tumors in (control *n* = 4, BRAFi *n* = 4, anti‐mVEGFA *n* = 4 and BRAFi + anti‐mVEGFA *n* = 4). (F) Immunofluorescence staining for and F4/80 (green) and MHCII (red) protein in D4M tumors treated with BRAFi in combination with anti‐VEGFA, (*n* = 3) Scale bar, 50 μm. (G) Representative flow cytometry plot and quantification of tumor infiltrating CD45^+^CD11b^+^Ly6C^hi^Ly6G^−^ monocytic myeloid‐derived suppressor cells (M‐MDSC; control *n* = 8, BRAFi *n* = 5, anti‐mVEGFA *n* = 7, BRAFi + anti‐mVEGFA *n* = 5) and CD45^+^CD11b^+^Ly6C^low^Ly6G^+^ PMN‐MDSC polymorphonuclear myeloid‐derived suppressor cells (PMN‐MDSC; control *n* = 8, BRAFi *n* = 5, anti‐mVEGFA *n* = 7, BRAFi + anti‐mVEGFA *n* = 5). Data are presented as means ± SEM. Significance was assessed by one‐way ANOVA test followed *post hoc* pairwise analysis test (A–D and G), *P < 0.05, **P < 0.01 and ***P < 0.001 vs control; ☨*P* < 0.05 versus BRAFi; ns, not significant.

To better investigate the role of BRAFi and its association with anti‐VEGFA antibody and understand the role exerted by recruited macrophages on shaping the immune response, we extended the observations raised in A375 model [24] in two immunocompetent melanoma models exploited by BRAFV600E mouse 5555 and D4M cells. In these syngeneic models, we explored the antitumor efficacy of short‐term administration (12 days) of BRAFi (PLX4720), anti‐mVEGFA (neutralizing antibody anti‐murine VEGFA; B20), and their combination therapy (herein after referred as VEGFA/BRAF targeting). In 5555 tumors, we observed that single‐agent BRAFi delayed tumor growth by 41%. However, 5555 tumors were completely refractory to anti‐VEGFA therapy and the association with VEGFA removal was not synergic (Fig. S1). In contrast, in D4M tumors, we observed BRAFi and anti‐mVEGFA antibody delayed tumor growth by around 45%, while their association substantially induced a 91% reduction in tumor volume as compared to untreated mice and a ~ 53% shrinkage of the initial tumor size (Fig. 2A). These data suggested that 5555 tumors were not sensitive to targeting VEGFA and we decided to proceed our investigation on D4M model.

First, we observed that microvessel area was significantly reduced by anti‐mVEGFA alone and by the dual therapy. Moreover, BRAFi significantly decreased the expression of HIF‐1α in D4M tumors, and the effect was maintained in combination with anti‐m‐VEGFA treatment but not after anti‐mVEGFA alone, indicating that BRAF inhibition is critical for abrogating tumor hypoxia (Fig. S2). We then assessed whether the combinatorial approach promoted the infiltration of tumor‐associated macrophages (TAMs) with a M1‐like phenotype. We observed that only the dual therapy induced an increase in CD68^+^ macrophage infiltration compared with untreated mice, while BRAFi treatment alone showed a modest but not significant inhibitory effect. Anti‐m‐VEGFA monotherapy did not affect macrophage infiltration (Fig. 2B). Flow cytometry analysis for macrophage population confirmed the immunofluorescence results and showed a significant increase in F4/80^+^ cells only after dual therapy (Fig. 2C). To confirm the TAM polarization state among the F4/80^+^ cell population, we analyzed by flow cytometry the surface expression of CD11c, a marker of M1‐like polarization [25]. We observed that BRAFi strongly led to the infiltration of CD11c^+^ M1‐like macrophages only when associated with anti‐mVEGFA and none of the treatments affected the infiltration of macrophages that express the M2 marker CD206 (Fig. 2D). In addition, sorted F4/80^+^ macrophage‐infiltrating D4M tumors treated by VEGFA/BRAF targeting showed increased transcripts of Tnfa, Cd86, Cxcl10, Cd40, Nos2, Cxcl9, Ccl5, which are recognized as markers of M1‐profile. At the same time, F4/80^+^ cells exhibited low levels of arginase (Arg1), one the marker associated with M2 polarization (Fig. 2E and Table S3). Immunofluorescence staining indicated also that macrophages infiltrating tumors co‐expressed MHC class II after dual therapy (Fig. 2F).

Tumors can also recruit MDSCs, which consist of two groups of cells termed polymorphonuclear (PMN‐MDSC) and monocytic (M‐MDSC) MDSCs. Accordingly, M‐MDSC and PMN‐MDSC can be defined as CD45^+^CD11b^+^Ly6C^hi^Ly6G^−^ and CD45^+^CD11b^+^Ly6C^low^Ly6G^+^, respectively [28]. We observed that dual therapy induced a statistically significant increment of tumor‐infiltrating M‐MDSCs compared with the control group, while BRAFi and anti‐m‐VEGFA single therapy did not alter the presence of M‐MDSCs. Moreover, anti‐mVEGFA monotherapy significantly increased the number of infiltrating PMN‐MDSCs but not when is associated with BRAFi (Fig. 2G).

### 3.3 GM‐CSF and infiltrating TAMs are dynamic biomarkers that correlate with response and relapse to dual anti‐mVEGFA and BRAFi therapy

**Fig. 3:**
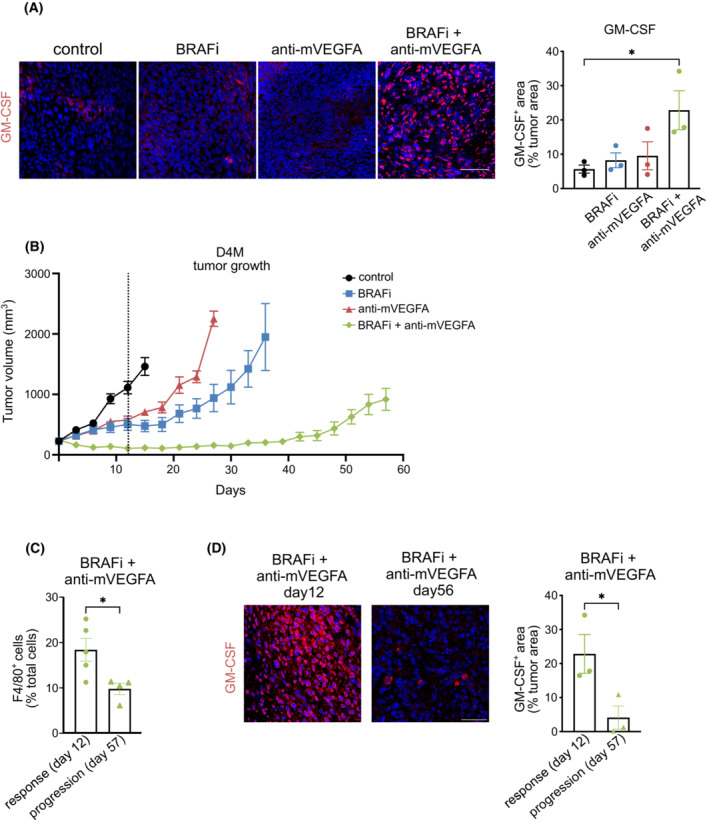
Antitumor activity induced by BRAF inhibitor (BRAFi) and anti‐murine VEGFA antibody (anti‐mVEGFA) combination therapy is correlated with macrophages infiltration and granulocyte‐macrophages colony‐stimulating factor (GM‐CSF) expression. (A) Representative images and quantification of GM‐CSF expression determined by immunofluorescence staining in D4M melanoma tumors treated as indicated. Bar graphs indicated the GM‐CSF^+^ area/tumor area, (control *n* = 3, BRAFi *n* = 3, anti‐mVEGFA *n* = 4, BRAFi + anti‐mVEGFA *n* = 3). Scale bar, 40 μm. (B) Mice bearing established D4M tumors were treated until tumors start to re‐growth (progression) with control (*n* = 7), BRAFi (PLX470; *n* = 7), anti‐m‐VEGFA (B20; *n* = 7) or BRAFi + anti‐m‐VEGFA (*n* = 8). Vertical broken line indicates the response phase (12 days of treatments). (C) Flow cytometry quantification of infiltrating CD45^+^F4/80^+^ macrophages are evaluated in D4M melanoma tumors treated with BRAFi + anti‐m‐VEGFA for 12 days (response phase; *n* = 5) or 57 days (progression phase; *n* = 4). (D) Representative images and quantification of GM‐CSF expression determined by immunofluorescence staining in D4M melanoma tumors treated with BRAFi + anti‐m‐VEGFA combination for 12 days (response phase; *n* = 3) or 57 days (progression phase; *n* = 3). Bar graphs indicated the GM‐CSF^+^ area/tumor area. Scale bar, 40 μm. Data are presented as means ± SEM. Significance was assessed by one‐way ANOVA test followed *post hoc* pairwise analysis test (A) and Student's *t*‐test (C, D), *P < 0.05.

We then evaluated the correlation between GM‐CSF expression and the recruited M1 macrophages in D4M tumors. Consistent with findings in the A375, immunofluorescence staining revealed a significant increase in GM‐CSF expression only after with the dual therapy compared with untreated mice, confirming the possible role of GM‐CSF in recruitment of M1 macrophages (Fig. 3A).

In order to explore the long‐term therapeutic effects of anti‐mVEGFA, BRAFi and their combination, we administered treatments over a period of 8 weeks. After 12 days of treatment, we distinguished a response phase, in which all treatments transiently reduced or blocked tumor growth, followed by a relapse phase, characterized by reinstatement of a robust tumor growth. We observed that tumor relapse progressively occurred after 27 and 36 days in mice that, respectively, received anti‐mVEGFA and BRAFi monotherapy. Nevertheless, the dual therapy substantially prolonged the progression‐free survival, showing a sustained tumor control. Despite the prolonged disease control, tumors treated with the dual therapy started to regrowth after 57 days (Fig. 3B). Melanoma tumors develop BRAFi resistance through a rewiring of signaling network that circumvents the BRAF blockade to achieve ERK activation. Here, we wanted to identify whether MAPK pathway is associated also with the acquisition of resistance to BRAF/VEGFA targeting. Interestingly, relapsed tumors after BRAFi or BRAFi + anti‐mVEGFA showed similar responses in terms of ERK reactivation as indicated by phopsho‐ERK immunofluorescence staining (Fig. S3A).

We then hypothesized that GM‐CSF expression and TAMs infiltration could represent key players in driving antitumor responses by dual therapy. To evaluate this correlation, we analyzed the number of infiltrating CD45^+^F4/80^+^ cells and GM‐CSF expression in D4M tumors at response and at progression. As shown in Fig. 3C, CD45^+^/F480^+^ cells were dramatically reduced in melanomas treated by long‐term dual regimen when the disease relapsed. Interestingly, the amount of GM‐CSF and the recruitment of M‐MDSC were diminished (Fig. 3D and Fig. S3B). On the opposite, the effect was not associated with the recruitment of PMN‐MDCs or a shift of the macrophage polarization phenotype, since PMN‐MDSCs and M2 macrophages are present in tumors in minor amount after 12 or 56 days of treatment (Fig. S3C,D).

### 3.4 Anti‐PD‐1 in association with BRAF/VEGFA blockade elicits durable responses and leads to tumor eradication

**Fig. 4:**
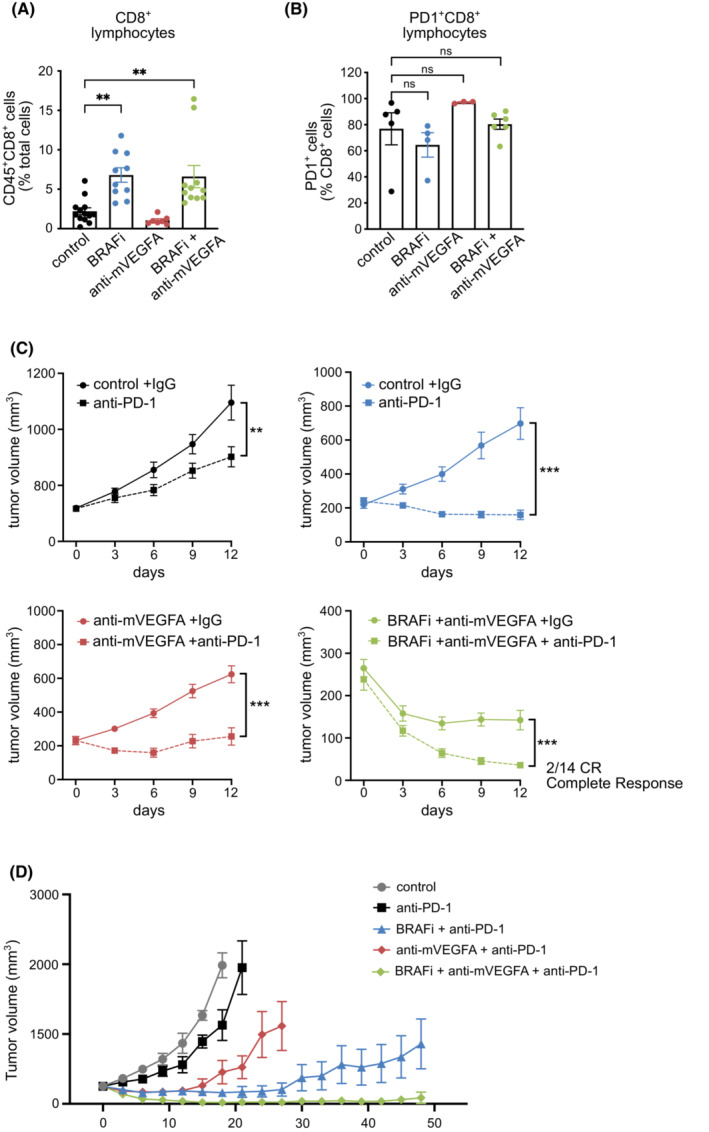
Anti‐PD‐1 enhances the efficacy of BRAF inhibitor (BRAFi), anti‐murine VEGFA antibody (anti‐mVEGFA) and their combination BRAFi, in D4M melanoma tumors. (A) Flow cytometry quantification of tumor infiltrating CD45^+^CD8^+^ T lymphocytes in D4M melanoma tumors in (control *n* = 13, BRAFi *n* = 10, anti‐mVEGFA *n* = 7, BRAFi + anti‐mVEGFA *n* = 11). (B) Flow cytometry quantification of the fraction of PD‐1 expression in tumor infiltrating CD8^+^ T lymphocytes in D4M melanoma tumors (control *n* = 5, BRAFi *n* = 4, anti‐mVEGFA *n* = 3, BRAFi + anti‐mVEGFA *n* = 6). (C) Mice bearing established D4M tumors were treated with isotype control or anti‐PD1 antibody (upper left panel), with BRAFi + isotype control or BRAFi + anti‐PD‐1 antibody (upper right panel), with anti‐m‐VEGFA + isotype control or anti‐m‐VEGFA + anti‐PD‐1 antibody (lower left panel), with BRAFi^+^ anti‐mVEGFA + isotype control or BRAFi + anti‐m‐VEGFA + anti‐PD‐1 antibody (lower right panel); (*n* = 7). (D) Mice bearing established D4M tumors were treated for 7 weeks with control (*n* = 7), anti‐PD‐1 (*n* = 7) BRAFi + anti‐PD‐1 (*n* = 6), anti‐mVEGFA + anti‐PD‐1 (*n* = 7) and BRAFi + anti‐mVEGFA + anti‐PD‐1 (*n* = 8). Data are presented as means ± SEM. Significance was assessed by one‐way ANOVA test followed *post hoc* pairwise analysis test (A, B) and Student's *t*‐test (C), **P < 0.01, ***P < 0.001; ns, not significant.

To investigate whether oncogenic BRAF targeting associated with VEGFA removal similarly modulate the adaptive immunity in D4M melanomas, we analyzed by flow cytometry the number of tumors infiltrating T cells after 12 days of treatment. Interestingly, we found that both BRAFi and dual therapy significantly increased CD8^+^ T‐cell tumor infiltration, while anti‐mVEGFA monotherapy did not influence their recruitment (Fig. 4A). Of note, none of the treatments affected the circulating fraction of CD8^+^ T lymphocytes in mice‐bearing D4M tumors (Fig. S4A). To better define the profile of infiltrating CD8^+^ lymphocytes, we evaluated the expression of the programmed cell death protein 1 receptor (PD‐1). As shown in Fig. 4B, PD‐1 was expressed by ~ 80% of intratumoral CD8^+^, and this phenotype was not significantly modified by drug treatments. In parallel to what observed for TAMs in D4M tumors (Fig. 3C), we found that the number of infiltrating CD8^+^ T cells dropped to levels comparable to untreated tumors during the relapse phase of the dual therapy (Fig. S4B).

The combined inhibition of BRAF and VEGFA reshapes the immunological landscape and therefore this condition may be exploited to improve the response to ICB. Mice‐bearing D4M tumors were concomitantly treated with anti‐PD‐1 antibody in different combinations with BRAFi and anti‐m‐VEGFA antibody for 12 days. Single anti‐PD‐1 antibody treatment moderately, but significantly, reduced tumor growth as compared to control antibody (Fig. 4C, left top panel). Nevertheless, combination of anti‐PD‐1 antibody with BRAFi or anti‐mVEGFA induced a significant reduction in tumor growth as compared to single BRAFi or anti‐mVEGFA alone (Fig. 4C, right top panel and left bottom panel). Combination of BRAFi and anti‐mVEGFA antibody showed the strongest efficacy in association with PD‐1 blockade (herein after referred as “triple therapy”) and resulted in tumor size reduction of 85% after 12 day of short‐term treatment. Interestingly, only triple therapy resulted in complete response (CR) in a subset of mice (2/14)‐bearing D4M melanomas (Fig. 4C, right bottom panel). We then assessed the efficacy of the long‐term treatments of the different therapeutic combinations in D4M melanomas. We observed that anti‐PD‐1 antibody enhanced the antitumor activity of anti‐mVEGFA and BRAFi, but the effect was temporarily limited. Long‐term treated mice with anti‐PD‐1 antibody in combination with anti‐mVEGFA antibody or BRAFi relapsed after 4 and 7 weeks of treatment, respectively. On the contrary, after 7 weeks, the triple therapy still induced a profound disease control with major tumor regression (70% of tumor volume inhibition from starting tumor size) (Fig. 4D). All the mice treated with triple regimen responded to therapy. In particular, 10 of 21 mice resulted in CR, six of 21 resulted in tumor volume regression from starting size (durable response, DR), and five of 21 responded but then progressed (short response, SR) (Fig. S4C). Notably, the triple therapy was well‐tolerated, as mice did not show any weight loss (Fig. S4D). In a second set of experiments in mice‐bearing D4M tumors, BRAFi and anti‐mVEGFA as single agents or in combination were maintained for 12 days and followed by anti‐PD‐1 antibody treatment. The sequential use of anti‐PD‐1 antibody did not improve tumor control and the tumors suddenly started to regrowth (Fig. S4E).

**Fig. 5:**
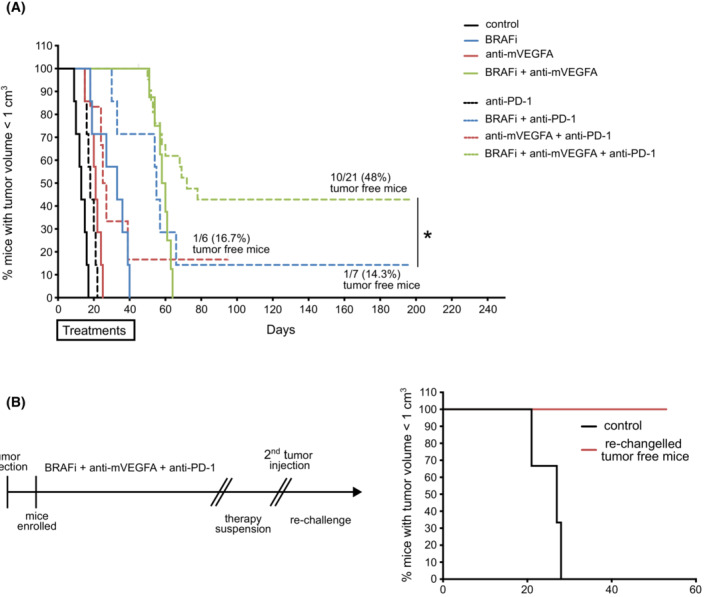
BRAF inhibitor (BRAFi) in association with anti‐murine VEGFA antibody (anti‐mVEGFA) and anti PD‐1 antibody (triple combination therapy) cures a large population of mice bearing D4M melanoma tumors and establishes protective memory in D4M tumor models. (A) Survival over time for mice bearing established D4M tumors. Tumor cell were subcutaneously inoculated in the right flank and treated 9 days after with BRAFi, anti‐m‐VEGFA, anti‐PD‐1 or the indicated double or triple combination: control (*n* = 7), BRAFi (*n* = 7), anti‐mVEGFA (*n* = 7), BRAFi + anti‐mVEGFA (*n* = 7) anti‐PD‐1 (*n* = 7) BRAFi + anti‐PD‐1 (*n* = 7), anti‐mVEGFA + anti‐PD‐1 (*n* = 6), BRAFi + anti‐mVEGFA + anti‐PD‐1 (*n* = 21). (B) Timeline of treatment (on the left) and survival over time for mice rechallenged with D4M tumor cells subcutaneous inoculation in the left flank (*n* = 6) (on the right). Significance was assessed by long‐rank (Mantel–Cox) test (A). *P < 0.05.

Of notice, the triple therapy resulted in CR in 48% mice (10 of 21) also after therapy suspension. The long‐term survivors lived up to 200 days since the termination of the therapy without any tumor mass (Fig. 5A) suggesting that the treatment had been curative. Doublet combinations between BRAFi or anti‐mVEGFA with antiPD‐1 elicited weaker therapeutic response and resulted in CR only in 14.3% (1 of 7) and 16.7% (1 of 6) mice, respectively. To assess immunologic memory response, cured mice after triplet suspension were then rechallenged on Day 96. D4M cells were injected in the opposite flank of the first injection, and naïve mice were challenged in parallel. We observed that no tumor growth occurred in 100% (6 of 6) of rechallenged mice, as well as no recurrence of the original tumor in the first side, demonstrating immunologic memory response after the rechallenge. In contrast, all naïve mice progressively developed tumors (Fig. 5B).

### 3.5 Response to triple therapy is mediated by CD8^+^ T cells and GM‐CSF recruited M1‐like macrophages

**Fig. 6:**
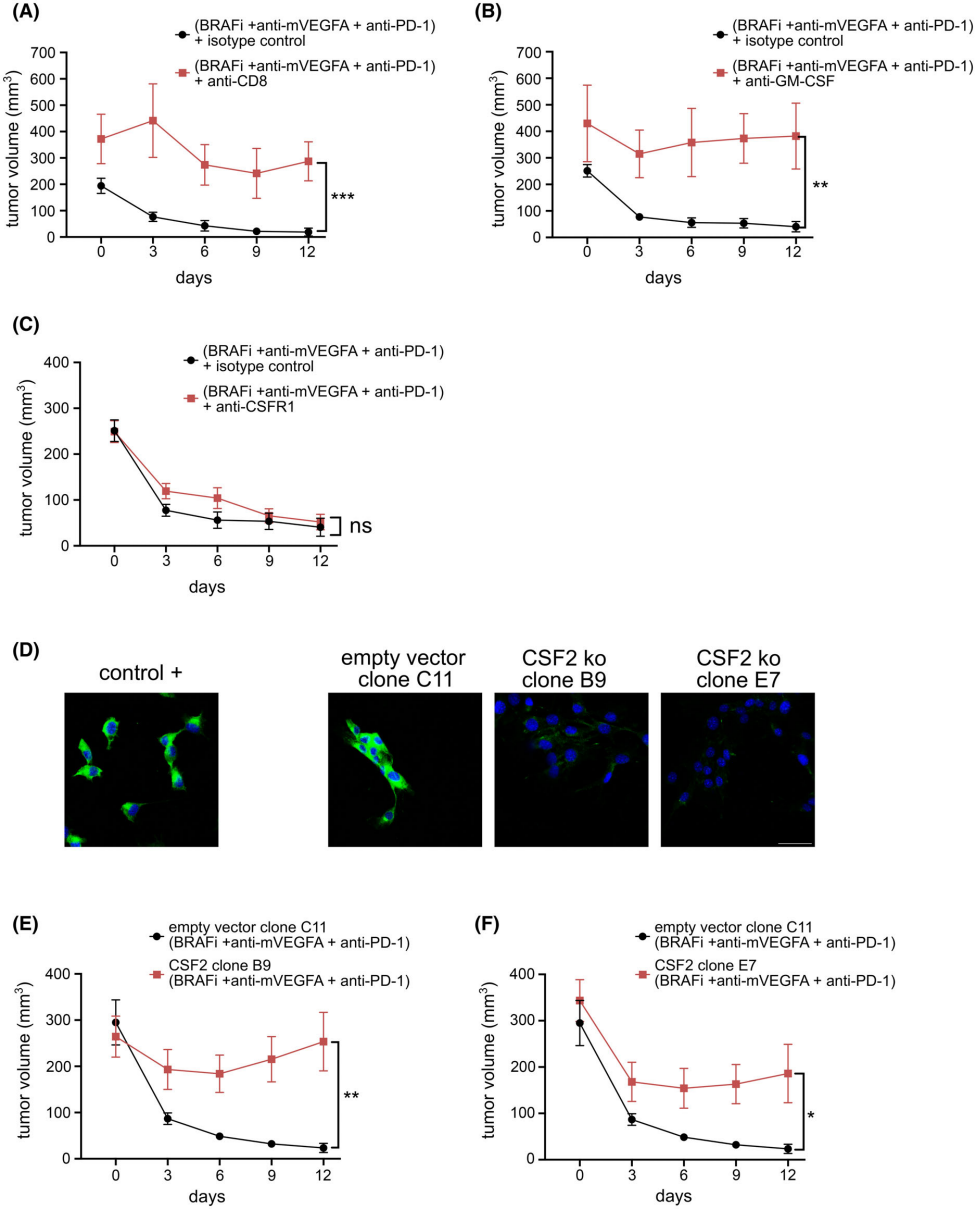
Granulocyte‐macrophages colony‐stimulating factor neutralization and genetic knock‐down demonstrates that tumor‐derived GM‐CSF regulates tumor clearing mechanism. (A) Mice bearing established D4M tumors were treated with BRAFi, anti‐mVEGFA and anti‐PD‐1 combination for 12 days with either a neutralizing anti‐CD8 antibody (*n* = 4) or isotype control antibody (*n* = 7), ***P < 0.001 versus control. (B) Mice bearing established D4M tumors were treated with BRAFi, anti‐mVEGFA and anti‐PD‐1 combination for 12 days with either a neutralizing anti‐GM‐CSF antibody (*n* = 4) or isotype control antibody (*n* = 7), **P < 0.01 versus control. (C) Mice bearing established D4M tumors were treated for with BRAFi, anti‐mVEGFA and anti‐PD‐1 combination for 12 days with either a neutralizing colony‐stimulating factor‐1 receptor antibody (anti‐CSFR1) (*n* = 8) or isotype control antibody (*n* = 7). (D) Representative images of GM‐CSF expression determined by immunofluorescence staining in a positive control, empty vector and GM‐CSF encoding gene, *Csf2* ko (clone B9 and clone E7) and D4M cells (*n* = 10). Scale bar, 40 μm. (E) C57BL/6 mice were implanted subcutaneously with either *Csf2* ko D4M cells, clone B9 (*n* = 6), or empty vector cells (*n* = 7). Mice bearing established D4M tumors were treated with BRAFi, anti‐mVEGFA and anti‐PD‐1 combination for 12 days. (F) C57BL/6 mice were implanted subcutaneously with either *Csf2* ko D4M cells, clone E7 (*n* = 6), or empty vector cells (*n* = 6). Mice bearing established D4M tumors were treated with BRAFi, anti‐mVEGFA and anti‐PD‐1 combination for 12 days. Data are presented as means ± SEM. Significance was assessed by Student's *t*‐test (A–C and E–F). *P < 0.05, **P < 0.01, ***P < 0.001; ns, not significant.

The above results raise the question whether CD8^+^ T cells and M1‐like macrophage infiltration are critical cellular players supporting the synergistic activity of BRAFi, VEGFA removal and PD‐1 blockade. To address this aspect, we depleted CD8^+^ cells in D4M melanomas. Mice with established tumors were then treated with the triplet for 12 days. We confirmed effective CD8^+^ cells suppression by analyzing circulating CD8^+^ cell population (Fig. S5A), and we reported that CD8^+^ depletion significantly enhanced tumor growth in untreated mice (Fig. S5B). Likewise, CD8^+^ cell depletion significantly impaired the antitumor activity upon triplet (Fig. 6A). According to previous data envisaging a role of GM‐CSF in recruiting M1‐like macrophages, we pretreated mice with neutralizing anti‐GM‐CSF antibody before starting the triple regimen. We observed that GM‐CSF blockade significantly enhanced tumor growth in untreated mice (Fig. S5C) and impaired the antitumor activity upon triple treatment with 11% vs 90% of tumor volume inhibition in presence of GM‐CSF neutralizing antibody or isotype‐matched immunoglobulins, respectively (Fig. 6B). Conversely, an antibody neutralizing mouse colony‐stimulating factor‐1 receptor (CSFR1), which is mainly expressed by M2‐like macrophages, did not interfere with the tumor growth both in untreated or in triple combination therapy‐treated mice (Fig. 6C and Fig. S5D).

Furthermore, we sought to understand the function of GM‐CSF produced by melanoma cells. To achieve this goal, we exploited genome editing with the CRISPR‐Cas9 system to inactivate GM‐CSF encoding gene, *Csf2*, in D4M cells. Because the expression of an exogenous Cas9 protein from *Streptococcus pyogenes* might affect cell growth in syngeneic mouse models by triggering immune‐mediated response [29], we generated *Csf2‐*knockout cell lines in which Cas9 was transiently expressed, as revealed by immunofluorescence staining (Fig. 6D and Fig. S5E). The knockout cell lines (clone B9 and E7) grew similarly to the empty vector controls (Fig. S5F,G). However, tumors derived from *Csf2‐*knockout B9 and E7 clones and treated for 12 days with the triple therapy showed a significant reduction in responsiveness to the therapy. As shown in Fig. 6E,F, triple therapeutic regimen reduced, respectively, of 92%, 4%, and 40% the tumor size of induced by empty‐vector control, B9 and E7 clones.

## 4 Discussion

Tumor‐associated macrophages play complex immunological roles in the TME, which vary with their activation state. Although M1/M2 macrophage polarization status is a simplification and cancer shows a broad variability in macrophages, their anti‐ and pro‐tumoral functions are paradigmatically connected to the classically activated (M1‐like) and alternatively activated (M2‐like) phenotype, respectively [29, 30]. Consequently, they have emerged as therapeutic targets in cancer therapy. Until today, macrophage‐targeting strategies mainly involve M2 TAM depletion or re‐education of the protumor M2 phenotype to M1‐like phenotype, crucial in inducing tumor regression [31]. However, increasing evidences indicate that M1‐like macrophages recruitment can limit tumor progression and support anticancer therapies. Our group, recently showed that the simultaneous BRAF/VEGFA targeting, induced the tumor infiltration of M1 macrophages, with a mandatory role in determining the delay of acquired resistance to BRAFi in a A375 melanoma model [24]. Here, we reported that BRAF/VEGFA targeting influenced monocyte‐derived macrophages infiltration through a cancer‐derived signal. For this, we used a new tool for the analyses of ligand–receptor interaction in the old A375 xenograft microarray dataset. We analyzed the human‐specific and mouse‐specific mRNAs differentially expressed after BRAF/VEGFA targeting in order to identify intercellular signals between cancer and stromal cells. This analysis identified a cytokine directly correlate with the recruitment in tumor of M1 macrophages, the GM‐CSF. In according to this, it has been demonstrated that GM‐CSF is a multipotent cytokine involved in monocyte recruitment and macrophage maturation and differentiation, regulating function as proliferation, phagocytosis, and antigen presentation [31]. However, conventional xenograft models have an immunocompromised status that is defective for adaptive immunity. Therefore, we exploited a syngeneic model to asses BRAF/VEGFA targeting in an immunocompetent model. In D4M tumors, we showed that the presence of T cells in TME does not hamper the recruitment of M1 macrophages and the synergistic antitumor activity induced by BRAF/VEGFA targeting. We showed that BRAF/VEGFA targeting increased the expression of costimulatory molecules, such as Cd40 and Cd86, in macrophages isolated from tumors. At the same time, macrophages that infiltrates in tumors after BRAF/VEGFA targeting show high expression of MHCII. MHCII expression in antigen presenting cells is a key regulator of adaptive immune response and, in agreement with our results, it has been shown that GM‐CSF treatment increased the expression of critical antigen‐presenting molecules such as HLA‐DR and CD86 in monocytes [32].

BRAF/VEGFA targeting increased also the infiltration of immature myeloid cells of monocyte origin, M‐MDSC, which can further mature and differentiate into M1 macrophages in the presence of GM‐CSF [33]. By contrast, anti‐VEGFA treatment alone mediated the recruitment of PMN‐MDSC. These data are in accordance with a published work that indicate that this cell population is associated with refractoriness to anti‐VEGFA therapy [34]. Nevertheless, we observed that the concomitant BRAF inhibition counteracts the PMN‐MDSC tumor infiltration induced by anti‐m‐VEGFA treatment.

We observed that the enhanced antitumor activity associated with the BRAF/VEGF targeting did not correlate with an increased inhibition of tumor angiogenesis. However, this report agrees with our previous works in which we confirmed that BRAFi abolished tumor tissue hypoxia in D4M tumors, also when coadministered with anti‐VEGFA [3, 24]. The restoration of the tissue oxygenation suggests the normalization of vessel functionality favoring the patrolling function of immune cells, as previously reported [10, 35].

The synergistic antitumor effect observed to BRAF/VEGFA targeting was transient. However, tumor recurrence was associated by a drop of M1 macrophages infiltration and GM‐CSF levels. Similarly, also the activation of the adaptive immune response was temporary. We observed that BRAFi induced the recruitment of CD8^+^ lymphocytes, even when associated with anti‐mVEGFA antibody. This is consistent with a previous work reporting that CD8^+^ cells increased following BRAFi but they disappeared at disease progression [5]. However, infiltrating CD8^+^ lymphocytes, driven in D4M tumors by BRAFi, showed an exhausted phenotype characterized by a high level of PD‐1 expression [36]. This observation suggests that the induction of PD‐1 on tumor‐infiltrating lymphocytes undermines their ability to mount an effective antitumor response and provides a plausible explanation for tumor growth despite the presence of infiltrating CD8^+^ cells.

Since innate immune cells can contribute to tumor suppression boosting a strong adaptive immune response, we hypothesized that infiltration on M1 macrophages in the TME by BRAF/VEGFA targeting would enhance the efficacy of the ICB, which primarily unleashes anticancer T‐cell response. In metastatic melanomas carrying BRAF, the combination of BRAFi with MEK inhibitor and immunotherapy represents a further step forward and a pilot clinical study suggests its clinical efficacy [8]. This study showed that a subset of patients (60%) had a long duration of response without evidence of acquired resistance to the oncogene‐targeted therapy at 2 years, but 40% of the patients relapsed, suggesting the need of a better selection of patients who are more likely to benefit from targeted therapies combined with immunotherapy.

Here, we demonstrated, in D4M tumors, VEGFA/BRAF targeting sensitized tumors to respond to anti‐PD1 therapy. Triple therapy induced a profound antitumor effect in all mice treated, without evidence of toxic effects. Notably, 50% of mice showed a complete tumor regression, even after therapy suspension and rechallenge with a second tumor injection, proving the activation of immunological memory. Clinical trials to determine the optimal schedule choice are ongoing (NC02224781). Nevertheless, we observed the sequential use of anti‐PD‐1 antibody, after 2 weeks of BRAF/VEGFA targeting, failed to improve tumor control and the tumors rapidly relapsed. This may indicate that control of tumor growth is dominated by targeted therapies and that a constant influence of the BRAF/VEGFA targeting on TME should be required to exploit the best efficacy of anti‐PD‐1 treatment. In according with our results, immune cell depletion studies combined with immunophenotypic analysis demonstrated that M1‐like TAM are required for synergistic curative activity of ICB associated with oncolytic viruses in glioblastoma [37]. Similarly, in hepatocellular carcinoma, Listeria‐based vaccine promotes M0 and M2 differentiation in M1‐TAM, which increased tumor PD‐L1 and improved the anti‐PD‐1 therapy [38]. Correlative studies conducted in cancer mouse models indicate that TLR7 agonist increases the ratio M1/M2 and dictates the improvement of the response to ICB [39]. Finally, PI3K inhibitors exploited in cancer treatment have been demonstrated to promote changes from M2 to M1 phenotype, synergizing with checkpoint inhibitors [40]. In D4M model, BRAFi alone was unable to recruit M1‐like macrophages and fully support ICB because only the removal of VEGFA allowed GM‐CSF expression, macrophage infiltration, and the improvement of immunotherapy effectiveness.

Finally, to validate the key role of the cooperation between GM‐CSF‐recruited M1 macrophages and activated CD8^+^ lymphocytes in orchestrating the eradication of tumors, we performed loss‐of‐function strategies in D4M model. We observed that the neutralization of GM‐CSF and CD8^+^ cell depletion inhibited similarly the efficacy of triple treatment. At the same way, triple therapy showed also poor effectiveness in tumors induced by *Csf2* null D4M cells. The role of GM‐CSF in addressing a therapeutic strategy in melanomas is supported by the clinical use of an oncolytic herpes virus vector encoding GM‐CSF. This vector reshapes the TME, increases CD8^+^ cell recruitment and tumor PD‐L1 expression, and induce a clinical response after subsequent ICB [41]. In agreement with TCGA data that indicates that 8% of mutated BRAFV600E human melanoma showed the overexpression of the VEGFA transcript, our work suggests that an improvement patient's molecular stratification taking into account the expression of VEGFA, might open new therapeutic regimens based on targeting VEGFA in advanced metastatic melanomas.

## 5 Conclusions

Our studies in melanoma tumors suggested that BRAF/VEGFA targeting reshaped the TME, leading to an improvement of anti‐PD‐1 effectiveness and inducing an immunologic memory response able to reject a second tumor. Our results imply that the efficacy of anti‐PD‐1 therapy depends mainly on the presence of M1‐like macrophages induced by GM‐CSF.

In summary, we have demonstrated that a rational combination of two targeted therapy (BRAFi and anti‐mVEGFA) and an immunomodulatory agent (anti‐PD‐1) in melanoma can lead to a CR in a large fraction of mice bearing melanomas not responding to BRAFi/anti‐PD‐1 combination therapy.

## Conflict of interest

AB declares the following competing interests: receipt of grants/research supports (Neophore, AstraZeneca, Boehringer), receipt of honoraria or consultation fees (Guardant Health, Inivata), Stock shareholder (Neophore, Kither), SAB (Inivata, Neophore, Roche/Genentech Global CRC Advisory Board) that are not related to the current study. FDN has received speaker's fees from Pierre Fabre, outside the submitted work. All the other authors have no conflicts of interest to declare.

## Author contributions

VC, FB, FDN, and DS conceived the study. VC, CG, VM, EV, and GD performed animal experiments. VC, VM, and GD performed immunophenotypic and immunohistochemistry analysis. SL performed gene‐editing experiments. CI and EM conducted bioinformatics data analyses. VC, FB, FDN, DS, and AB interpreted the data. VC, FB, FDN, DS, and AB wrote the paper. FB and FDN coordinate the study.

### Peer review

The peer review history for this article is available at https://www.webofscience.com/api/gateway/wos/peer‐review/10.1002/1878‐0261.13450.

## Supporting information


**Fig. S1.** Syngeneic 5555 melanoma tumors are refractory to anti‐mVEGFA antibody and BRAF/VEGFA targeting does not result in synergistic antitumor activity.
**Fig. S2.** Synergistic antitumor activity induced by BRAF/VEGFA targeting is not correlated with an augmented inhibition of tumor angiogenesis in D4M syngeneic melanoma model.
**Fig. S3.** BRAF/VEGFA targeting delays the onset to acquired resistance to BRAFi in D4M syngeneic melanoma model.
**Fig. S4.** Anti‐PD‐1 enhances the efficacy of BRAFi, anti‐m‐VEGFA, and their combination in D4M syngeneic melanoma model.
**Fig. S5.** GM‐CSF neutralization and genetic knockdown demonstrates that tumor‐derived GM‐CSF regulates tumor‐clearing mechanism in D4M syngeneic melanoma model.

**Table S1.** Differential expression of ligand upon treatments including p value, BH adjusted, and log2ratio comparing each condition against the controls.

**Table S2.** Differential expression of receptor upon treatments including p value, BH adjusted, and log2ratio comparing each condition against the controls.

**Table S3.** Log2 Fold Change Real‐Time PCR analysis of macrophages polarization.

## Data Availability

The raw research data are available per request through the corresponding author.

## References

[1] Luke JJ , Flaherty KT , Ribas A , Long GV . Targeted agents and immunotherapies: optimizing outcomes in melanoma. Nat Rev Clin Oncol. 2017;14(8):463–82. 28374786 10.1038/nrclinonc.2017.43

[2] Murciano‐Goroff YR , Warner AB , Wolchok JD . The future of cancer immunotherapy: microenvironment‐targeting combinations. Cell Res. 2020;30(6):507–19. 32467593 10.1038/s41422-020-0337-2 PMC7264181

[3] Bottos A , Martini M , Di Nicolantonio F , Comunanza V , Maione F , Minassi A , et al. Targeting oncogenic serine/threonine‐protein kinase BRAF in cancer cells inhibits angiogenesis and abrogates hypoxia. Proc Natl Acad Sci USA. 2012;109(6):E353–9. 10.1073/pnas.1105026109 22203991 PMC3277561

[4] Kuske M , Westphal D , Wehner R , Schmitz M , Beissert S , Praetorius C , et al. Immunomodulatory effects of BRAF and MEK inhibitors: implications for melanoma therapy. Pharmacol Res. 2018;136:151–9. 30145328 10.1016/j.phrs.2018.08.019

[5] Wilmott JS , Long GV , Howle JR , Haydu LE , Sharma RN , Thompson JF , et al. Selective BRAF inhibitors induce marked T‐cell infiltration into human metastatic melanoma. Clin Cancer Res. 2012;18(5):1386–94. 22156613 10.1158/1078-0432.CCR-11-2479

[6] Frederick DT , Piris A , Cogdill AP , Cooper ZA , Lezcano C , Ferrone CR , et al. BRAF inhibition is associated with enhanced melanoma antigen expression and a more favorable tumor microenvironment in patients with metastatic melanoma. Clin Cancer Res. 2013;19(5):1225–31. 23307859 10.1158/1078-0432.CCR-12-1630 PMC3752683

[7] Hu‐Lieskovan S , Mok S , Homet Moreno B , Tsoi J , Robert L , Goedert L , et al. Improved antitumor activity of immunotherapy with BRAF and MEK inhibitors in *BRAF* ^ *V600E* ^ melanoma. Sci Transl Med. 2015;7(279):279ra41. 10.1126/scitranslmed.aaa4691 PMC4765379 25787767

[8] Ribas A , Lawrence D , Atkinson V , Agarwal S , Miller WH , Carlino MS , et al. Combined BRAF and MEK inhibition with PD‐1 blockade immunotherapy in BRAF‐mutant melanoma. Nat Med. 2019;25(6):936–40. 31171879 10.1038/s41591-019-0476-5 PMC8562134

[9] Ascierto PA , Ferrucci PF , Fisher R , Del Vecchio M , Atkinson V , Schmidt H , et al. Dabrafenib, trametinib and pembrolizumab or placebo in BRAF‐mutant melanoma. Nat Med. 2019;25(6):941–6. 31171878 10.1038/s41591-019-0448-9

[10] Motz GT , Santoro SP , Wang LP , Garrabrant T , Lastra RR , Hagemann IS , et al. Tumor endothelium FasL establishes a selective immune barrier promoting tolerance in tumors. Nat Med. 2014;20(6):607–15. 24793239 10.1038/nm.3541 PMC4060245

[11] Ohm JE , Gabrilovich DI , Sempowski GD , Kisseleva E , Parman KS , Nadaf S , et al. VEGF inhibits T‐cell development and may contribute to tumor‐induced immune suppression. Blood. 2003;101(12):4878–86. 12586633 10.1182/blood-2002-07-1956

[12] Voron T , Colussi O , Marcheteau E , Pernot S , Nizard M , Pointet AL , et al. VEGF‐A modulates expression of inhibitory checkpoints on CD8+ T cells in tumors. J Exp Med. 2015;212(2):139–48. 25601652 10.1084/jem.20140559 PMC4322048

[13] Gabrilovich D , Ishida T , Oyama T , Ran S , Kravtsov V , Nadaf S , et al. Vascular endothelial growth factor inhibits the development of dendritic cells and dramatically affects the differentiation of multiple hematopoietic lineages in vivo. Blood. 1998;92(11):4150–66. 9834220

[14] Huang Y , Chen X , Dikov MM , Novitskiy SV , Mosse CA , Yang L , et al. Distinct roles of VEGFR‐1 and VEGFR‐2 in the aberrant hematopoiesis associated with elevated levels of VEGF. Blood. 2007;110(2):624–31. 17376891 10.1182/blood-2007-01-065714 PMC1924481

[15] Dirkx AEM , Heijnen VVT . Tumor angiogenesis modulates leukocyte‐vessel wall interactions in vivo by reducing endothelial adhesion molecule expression. Cancer Res. 2003;63:2322–9. 12727857

[16] Apte RS , Chen DS , Ferrara N . VEGF in signaling and disease: beyond discovery and development. Cell. 2019;176(6):1248–64. 30849371 10.1016/j.cell.2019.01.021 PMC6410740

[17] Hodi FS , Lawrence D , Lezcano C , Wu X , Zhou J , Sasada T , et al. Bevacizumab plus ipilimumab in patients with metastatic melanoma. Cancer Immunol Res. 2014;2(7):632–42. 24838938 10.1158/2326-6066.CIR-14-0053 PMC4306338

[18] Huang Y , Yuan J , Righi E , Kamoun WS , Ancukiewicz M , Nezivar J , et al. Vascular normalizing doses of antiangiogenic treatment reprogram the immunosuppressive tumor microenvironment and enhance immunotherapy. Proc Natl Acad Sci USA. 2012;109(43):17561–6. 23045683 10.1073/pnas.1215397109 PMC3491458

[19] Schmittnaegel M , Rigamonti N , Kadioglu E , Cassará A , Wyser Rmili C , Kiialainen A , et al. Dual angiopoietin‐2 and VEGFA inhibition elicits antitumor immunity that is enhanced by PD‐1 checkpoint blockade. Sci Transl Med. 2017;9(385):eaak9670. 28404865 10.1126/scitranslmed.aak9670

[20] Osada T , Chong G , Tansik R , Hong T , Spector N , Kumar R , et al. The effect of anti‐VEGF therapy on immature myeloid cell and dendritic cells in cancer patients. Cancer Immunol Immunother. 2008;57(8):1115–24. 18193223 10.1007/s00262-007-0441-x PMC4110970

[21] Ugurel S , Rappl G , Tilgen W , Reinhold U . Increased serum concentration of angiogenic factors in malignant melanoma patients correlates with tumor progression and survival. J Clin Oncol. 2001;19(2):577–83. 11208853 10.1200/JCO.2001.19.2.577

[22] Yuan J , Zhou J , Dong Z , Tandon S , Kuk D , Panageas KS , et al. Pretreatment serum VEGF is associated with clinical response and overall survival in advanced melanoma patients treated with ipilimumab. Cancer Immunol Res. 2014;2(2):127–32. 24778276 10.1186/2051-1426-1-S1-P247 PMC3991109

[23] Socinski MA , Jotte RM , Cappuzzo F , Orlandi F , Stroyakovskiy D , Nogami N , et al. Atezolizumab for first‐line treatment of metastatic nonsquamous NSCLC. N Engl J Med. 2018;378(24):2288–301. 29863955 10.1056/NEJMoa1716948

[24] Comunanza V , Corà D , Orso F , Consonni FM , Middonti E , Di Nicolantonio F , et al. vegf blockade enhances the antitumor effect of braf ^ v 600E^ inhibition. EMBO Mol Med. 2017;9(2):219–37. 27974353 10.15252/emmm.201505774 PMC5286370

[25] Jenkins MH , Steinberg SM , Alexander MP , Fisher JL , Ernstoff MS , Turk MJ , et al. Multiple murine BRaf ^V600E^ melanoma cell lines with sensitivity to PLX4032. Pigment Cell Melanoma Res. 2014;27(3):495–501. 24460976 10.1111/pcmr.12220 PMC3988244

[26] Dhomen N , Reis‐Filho JS , da Rocha DS , Hayward R , Savage K , Delmas V , et al. Oncogenic Braf induces melanocyte senescence and melanoma in mice. Cancer Cell. 2009;15(4):294–303. 19345328 10.1016/j.ccr.2009.02.022

[27] Isella C , Terrasi A , Bellomo SE , Petti C , Galatola G , Muratore A , et al. Stromal contribution to the colorectal cancer transcriptome. Nat Genet. 2015;47(4):312–9. 25706627 10.1038/ng.3224

[28] Brancato V , Comunanza V , Imparato G , Corà D , Urciuolo F , Noghero A , et al. Bioengineered tumoral microtissues recapitulate desmoplastic reaction of pancreatic cancer. Acta Biomater. 2017;49:152–66. 27916739 10.1016/j.actbio.2016.11.072

[29] Noy R , Pollard JW . Tumor‐associated macrophages: from mechanisms to therapy. Immunity. 2014;41(1):49–61. 25035953 10.1016/j.immuni.2014.06.010 PMC4137410

[30] Gabrilovich DI . Myeloid‐derived suppressor cells. Cancer Immunol Res. 2017;5(1):3–8. 28052991 10.1158/2326-6066.CIR-16-0297 PMC5426480

[31] Locati M , Curtale G , Mantovani A . Diversity, mechanisms, and significance of macrophage plasticity. Annu Rev Pathol. 2020;15(1):123–47. 31530089 10.1146/annurev-pathmechdis-012418-012718 PMC7176483

[32] Mantovani A , Allavena P , Marchesi F , Garlanda C . Macrophages as tools and targets in cancer therapy. Nat Rev Drug Discov. 2022;21(11):799–820. 35974096 10.1038/s41573-022-00520-5 PMC9380983

[33] Wicks IP , Roberts AW . Targeting GM‐CSF in inflammatory diseases. Nat Rev Rheumatol. 2016;12(1):37–48. 26633290 10.1038/nrrheum.2015.161

[34] Lotfi N , Zhang GX , Esmaeil N , Rostami A . Evaluation of the effect of GM‐CSF blocking on the phenotype and function of human monocytes. Sci Rep. 2020;10(1):1567. 32005854 10.1038/s41598-020-58131-2 PMC6994676

[35] Van Overmeire E , Stijlemans B , Heymann F , Keirsse J , Morias Y , Elkrim Y , et al. M‐CSF and GM‐CSF receptor signaling differentially regulate monocyte maturation and macrophage polarization in the tumor microenvironment. Cancer Res. 2016;76(1):35–42. 26573801 10.1158/0008-5472.CAN-15-0869

[36] Shojaei F , Wu X , Malik AK , Zhong C , Baldwin ME , Schanz S , et al. Tumor refractoriness to anti‐VEGF treatment is mediated by CD11b+Gr1+ myeloid cells. Nat Biotechnol. 2007;25(8):911–20. 17664940 10.1038/nbt1323

[37] Motz GT , Coukos G . Deciphering and reversing tumor immune suppression. Immunity. 2013;39(1):61–73. 23890064 10.1016/j.immuni.2013.07.005 PMC3782392

[38] Ahmadzadeh M , Johnson LA , Heemskerk B , Wunderlich JR , Dudley ME , White DE , et al. Tumor antigen–specific CD8 T cells infiltrating the tumor express high levels of PD‐1 and are functionally impaired. Blood. 2009;114(8):1537–44. 19423728 10.1182/blood-2008-12-195792 PMC2927090

[39] Saha D , Martuza RL , Rabkin SD . Macrophage polarization contributes to glioblastoma eradication by combination immunovirotherapy and immune checkpoint blockade. Cancer Cell. 2017;32(2):253–67.e5. 28810147 10.1016/j.ccell.2017.07.006 PMC5568814

[40] Xu G , Feng D , Yao Y , Li P , Sun H , Yang H , et al. Listeria‐based hepatocellular carcinoma vaccine facilitates anti‐PD‐1 therapy by regulating macrophage polarization. Oncogene. 2020;39(7):1429–44. 31659256 10.1038/s41388-019-1072-3

[41] Sato‐Kaneko F , Yao S , Ahmadi A , Zhang SS , Hosoya T , Kaneda MM , et al. Combination immunotherapy with TLR agonists and checkpoint inhibitors suppresses head and neck cancer. JCI Insight. 2017;2(18):e93397. 28931759 10.1172/jci.insight.93397 PMC5621908

