# Supplementary material for: Dual VEGFA/BRAF targeting boosts PD‐1 blockade in melanoma through GM‐CSF‐mediated infiltration of M1 macrophages
Source: Mol Oncol. 2023 May 27;17(8):1474–91. doi: 10.1002/1878-0261.13450 (PMC10399721; doi:10.1002/1878-0261.13450)
Supplement: Supplementary file 1 — Fig. S1. Syngeneic 5555 melanoma tumors are refractory to anti‐mVEGFA antibody and BRAF/VEGFA targeting does not result in synergistic antitumor activity. Fig. S2. Synergistic antitumor activity induced by BRAF/VEGFA targeting is not correlated with an augmented inhibition of tumor angiogenesis in D4M syngeneic melanoma model. Fig. S3. BRAF/VEGFA targeting delays the onset to acquired resistance to BRAFi in D4M syngeneic melanoma model. Fig. S4. Anti‐PD‐1 enhances the efficacy of BRAFi, anti‐m‐VEGFA, and their combination in D4M syngeneic melanoma model. Fig. S5. GM‐CSF neutralization and genetic knockdown demonstrates that tumor‐derived GM‐CSF regulates tumor‐clearing mechanism in D4M syngeneic melanoma model. [file MOL2-17-1474-s001.zip › FiguresS1-S5_Legends.docx]

Dual VEGFA/BRAF targeting boosts PD-1 blockade in melanoma through GM-CSF-mediated infiltration of M1-macrophages

Valentina Comunanza^1,2^ *, Chiara Gigliotti^1,2^, Simona Lamba^2^, Gabriella Doronzo^1,2^ Edoardo Vallariello^1,2,^ Valentina Martin^2^, Claudio Isella^1,2^, Enzo Medico^1,2^, Alberto Bardelli^1,2,3^ Dario Sangiolo^1,2^, Federica Di Nicolantonio^1,2 †^, Federico Bussolino^1,2^ *^†^

Supporting Information

**Supplemental Figure Legends**

**Supplementary Figure 1.** Syngeneic 5555 melanoma tumors are refractory to anti-murine vascular endothelial growth factor antibody (anti-mVEGFA) and BRAF/VEGFA targeting does not result in synergistic anti-tumor activity. Mice bearing established 5555 tumors were treated for 12 days with control (n = 14), BRAF inhibitor (BRAFi; PLX470; n =14), anti-mVEGFA (B20; n = 14) or BRAFi + anti-mVEGFA (n = 14).

**Supplementary Figure 2.** Synergistic antitumor activity induced by BRAF/VEGFA targeting is not correlated with an augmented inhibition of tumor angiogenesis in D4M syngeneic melanoma model.

Representative images and quantification of vasculature and hypoxia inducible factor 1-α (HIF-1α) expression determined by CD31 (*green*) and HIF-1α (*red*) immunofluorescence staining in D4M melanoma tumors treated as indicated: control (n = 4), BRAF inhibitor (BRAFi; n = 4), anti-murine vascular endothelial growth factor antibody (anti-mVEGFA; n = 3), BRAFi + anti-mVEGFA (n = 3). Scale bar, 50 μm. Quantification of tumor vasculature was determined as microvessel area (MVA) by CD31 immunofluorescence staining. Data are presented as means ± SEM. Significance was assessed by one-way ANOVA test followed *post hoc* pairwise analysis test, *P < 0.05.

**Supplementary Figure 3.** BRAF/VEGFA targeting delays the onset to acquired resistance to BRAF inhibitor (BRAFi) in D4M syngeneic melanoma model. (A) Representative images and quantification of pERK immunofluorescence staining in D4M melanoma tumors treated as indicated. Bar graphs indicated the pERK^+^/DAPI^+^ area/tumor area in control (n = 3) or BRAFi (n = 3), anti-murine vascular endothelial growth factor antibody (anti-mVEGFA; n = 3), BRAFi + anti-mVEGFA (n = 3) treated tumors. Scale bar, 40 μm. (B) Quantification of tumor infiltrating CD45^+^CD11b^+^Ly6ChiLy6G^-^ monocytic myeloid-derived suppressor cells (M-MDSC; in D4M melanoma tumors in control (n =8) or treated with BRAFi for 12 days (response; n = 5) or 36 days (progression; n = 4), anti-mVEGFA for 12 days (response; n = 7) or 27days (progression; n = 4), and BRAFi + anti-mVEGFA for 12 days (response; n = 5) or 57 days (progression; n = 3). (C) Quantification of tumor infiltrating CD45^+^CD11b^+^Ly6C^low^Ly6G^+^ polymorphonuclear myeloid-derived suppressor cells (PMN-MDSC) in D4M melanoma tumors in control (n = 8) or treated with BRAFi for 12 days (response; n = 6) or 36 days (progression; n = 4), anti-mVEGFA for 12 days (response; n = 7) or 27 days (progression; n = 4), and BRAFi + anti-mVEGFA for 12 days (response; n = 7) or 57 days (progression; n =4). (D) Quantification of tumor infiltrating F4/80^+^CD206^+^ M2-macrophages in D4M melanoma tumors treated with BRAFi + anti-mVEGFA for 12 days (response; n = 6) or 57 days (progression; n =4), Data are presented as means ± SEM. Significance was assessed and Student’s t-test (A-C). **P* < 0.05, ***P* < 0.01.

**Supplementary Figure 4.** Anti-PD-1 enhances the efficacy of BRAF inhibitor (BRAFi), anti-murine vascular endothelial growth factor antibody (anti-mVEGFA) and their combination in D4M syngeneic melanoma model. (A) Flow-cytometry quantification of circulating CD45^+^CD8^+^ T lymphocytes in mice bearing D4M tumors in control (n = 12) and after treatment with BRAFi (n = 5), anti-mVEGFA (n = 6) and BRAFi + anti-mVEGFA (n = 6). (B) Flow-cytometry quantification of infiltrating CD45^+^CD8^+^ T lymphocytes in mice bearing D4M tumors treated with and BRAFi + anti-mVEGFA for 12 days (response; n = 9) or 57 days (progression; n = 3). (C) Tumor growth curves from mice bearing D4M tumors and treated with triple combination therapy (BRAFi + anti-mVEGFA + anti-PD-1) for 7 weeks defines three cohorts: CR (complete response, n = 10), DR (durable response, n = 6) and short response (SR, n = 5). (D) Mice bearing established D4M tumors were monitored for body weight during treatment with triple combination therapy (BRAFi + established D4M anti-mVEGF-A + anti-PD-1). (E) Mice bearing established D4M tumors were treated for 12 days with targeted therapy: control (n = 7), BRAFi (n = 7), anti-mVEGF-A (n = 7) and BRAFi + anti-mVEGF-A (n = 7). At day 12 targeted therapies were suspended and mice of all groups were treated for 15 days with and anti-PD-1 antibody. Data are presented as means ± SEM. Significance was assessed by Student’s t-test (B). ***P < 0.001.

**Supplementary Figure 5.** Granulocyte-macrophages colony-stimulating factor (GM-CSF) neutralization and genetic knock-down demonstrates that tumor-derived GM-CSF regulates tumor clearing mechanism in D4M syngeneic melanoma model. (A) Representative flow plot of circulating infiltrating CD45^+^CD3^+^CD8^+^ T lymphocytes evaluated by flow cytometry in D4M tumors treated for 12 days with a neutralizing anti-CD8 antibody or isotype control antibody (n = 4). Quadrants were set based on isotype control antibody. (B) Mice bearing established D4M tumors were treated for 12 days with a neutralizing anti-CD8 antibody (n = 4) or isotype control antibody (n = 7). (C) Mice bearing established D4M tumors were treated for 12 days with a neutralizing anti-GM-CSF antibody (n = 4) or isotype control antibody (n = 7). (D) Mice bearing established D4M tumors were treated for 12 days with a neutralizing colony-stimulating factor-1 receptor antibody (anti-CSFR1) (n = 8) or isotype control antibody (n = 7). (E) Representative images of CAS9 expression determined by immunofluorescence staining in a positive control, GM-CSF encoding gene*,* (*Csf2)* ko (clone B9 and clone E7) and empty vector D4M cells (n = 10). Scale bar, 40 μm. (F) C57BL/6 mice were implanted subcutaneously with either *Csf2* ko D4M cells, clone B9 (n = 7), or empty vector cells (n = 7). (G) C57BL/6 mice were implanted subcutaneously with either *Csf2* ko D4M cells, clone E7 (n = 7), or empty vector cells (n = 7). Data are presented as means ± SEM. Significance was assessed and Student’s t-test (B-C). *P < 0.05, *P < 0.01.
