# Supplementary figures and images for: Dual VEGFA/BRAF targeting boosts PD‐1 blockade in melanoma through GM‐CSF‐mediated infiltration of M1 macrophages
Source: Mol Oncol. 2023 May 27;17(8):1474–91. doi: 10.1002/1878-0261.13450 (PMC10399721; doi:10.1002/1878-0261.13450)

5555  
tumor growth

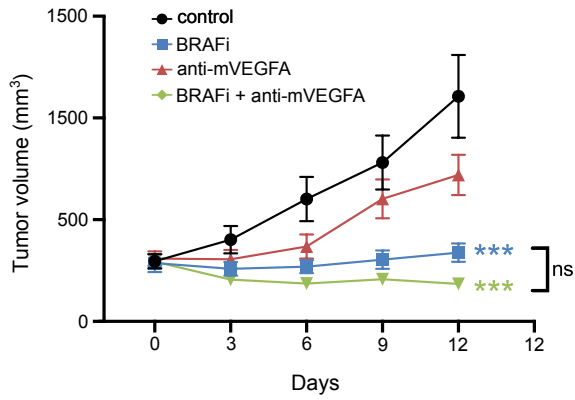

Supplement: Supplementary file 1 — Fig. S1. Syngeneic 5555 melanoma tumors are refractory to anti‐mVEGFA antibody and BRAF/VEGFA targeting does not result in synergistic antitumor activity. Fig. S2. Synergistic antitumor activity induced by BRAF/VEGFA targeting is not correlated with an augmented inhibition of tumor angiogenesis in D4M syngeneic melanoma model. Fig. S3. BRAF/VEGFA targeting delays the onset to acquired resistance to BRAFi in D4M syngeneic melanoma model. Fig. S4. Anti‐PD‐1 enhances the efficacy of BRAFi, anti‐m‐VEGFA, and their combination in D4M syngeneic melanoma model. Fig. S5. GM‐CSF neutralization and genetic knockdown demonstrates that tumor‐derived GM‐CSF regulates tumor‐clearing mechanism in D4M syngeneic melanoma model. [file MOL2-17-1474-s001.zip › Figure S1.pdf]

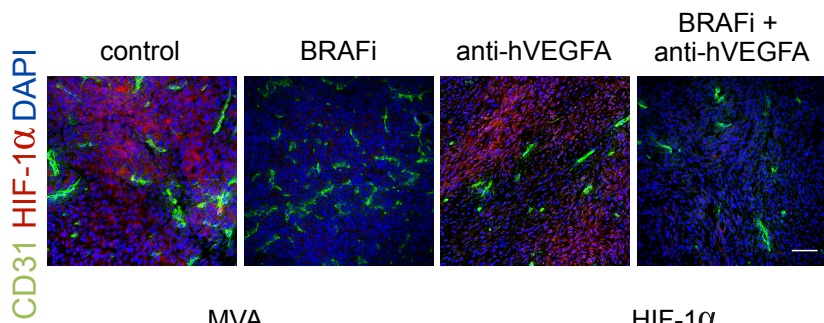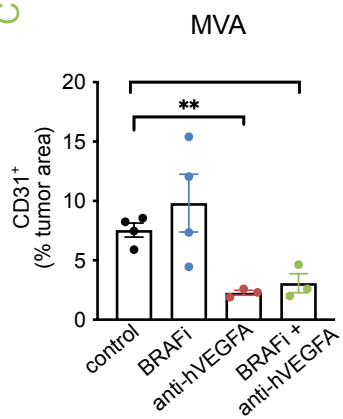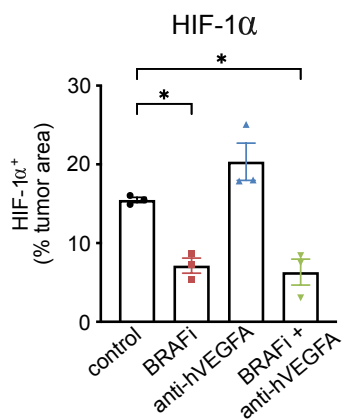

Supplement: Supplementary file 1 — Fig. S1. Syngeneic 5555 melanoma tumors are refractory to anti‐mVEGFA antibody and BRAF/VEGFA targeting does not result in synergistic antitumor activity. Fig. S2. Synergistic antitumor activity induced by BRAF/VEGFA targeting is not correlated with an augmented inhibition of tumor angiogenesis in D4M syngeneic melanoma model. Fig. S3. BRAF/VEGFA targeting delays the onset to acquired resistance to BRAFi in D4M syngeneic melanoma model. Fig. S4. Anti‐PD‐1 enhances the efficacy of BRAFi, anti‐m‐VEGFA, and their combination in D4M syngeneic melanoma model. Fig. S5. GM‐CSF neutralization and genetic knockdown demonstrates that tumor‐derived GM‐CSF regulates tumor‐clearing mechanism in D4M syngeneic melanoma model. [file MOL2-17-1474-s001.zip › Figure S2.pdf]

**A**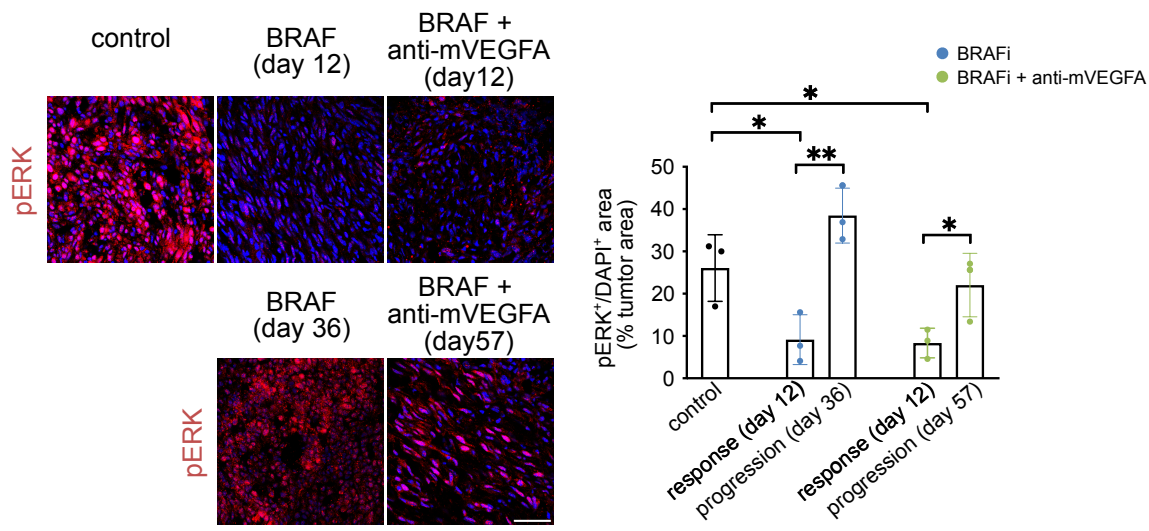**B**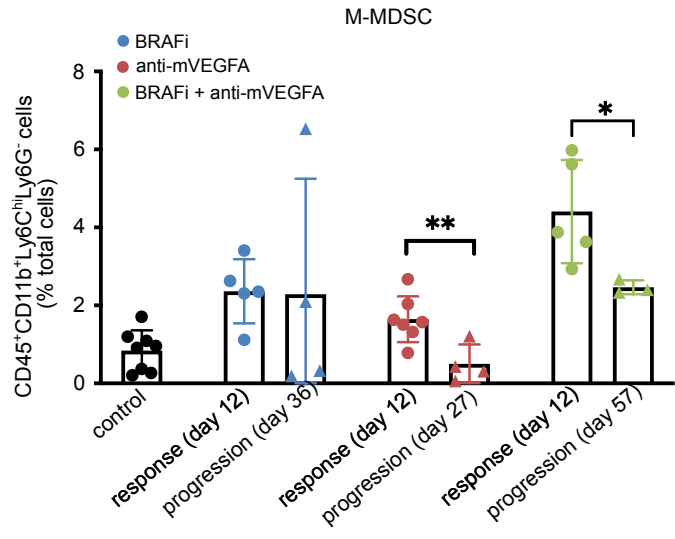**C**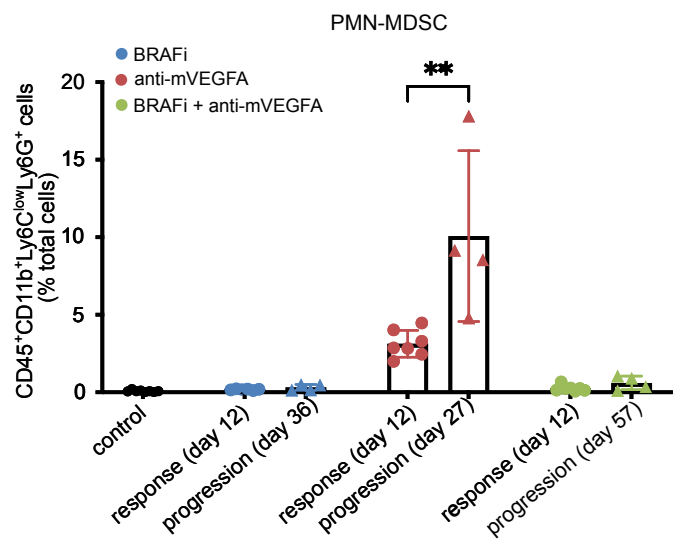**D**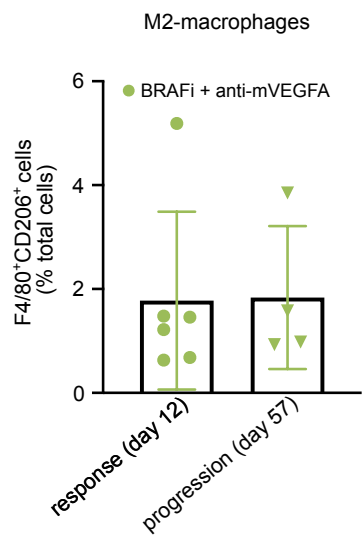

Supplement: Supplementary file 1 — Fig. S1. Syngeneic 5555 melanoma tumors are refractory to anti‐mVEGFA antibody and BRAF/VEGFA targeting does not result in synergistic antitumor activity. Fig. S2. Synergistic antitumor activity induced by BRAF/VEGFA targeting is not correlated with an augmented inhibition of tumor angiogenesis in D4M syngeneic melanoma model. Fig. S3. BRAF/VEGFA targeting delays the onset to acquired resistance to BRAFi in D4M syngeneic melanoma model. Fig. S4. Anti‐PD‐1 enhances the efficacy of BRAFi, anti‐m‐VEGFA, and their combination in D4M syngeneic melanoma model. Fig. S5. GM‐CSF neutralization and genetic knockdown demonstrates that tumor‐derived GM‐CSF regulates tumor‐clearing mechanism in D4M syngeneic melanoma model. [file MOL2-17-1474-s001.zip › Figure S3.pdf]

A

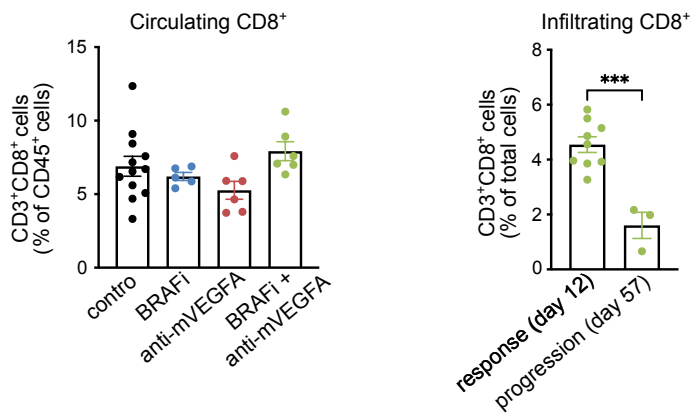

C

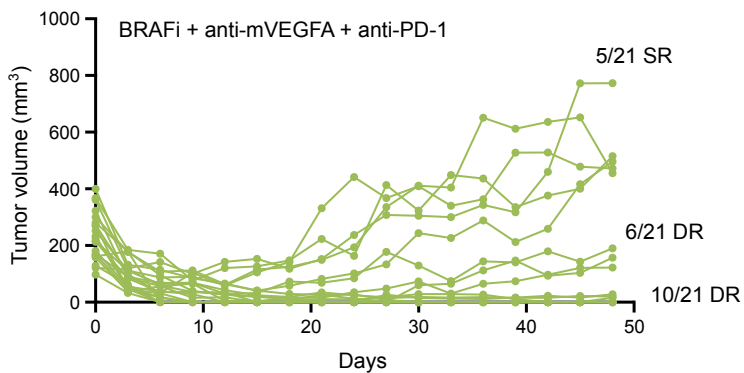

D

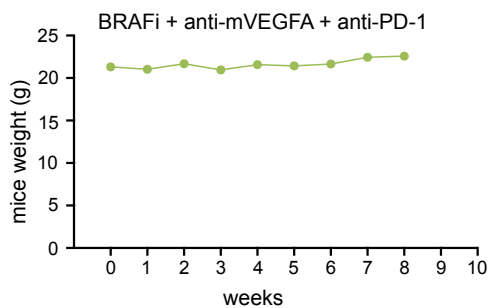

E

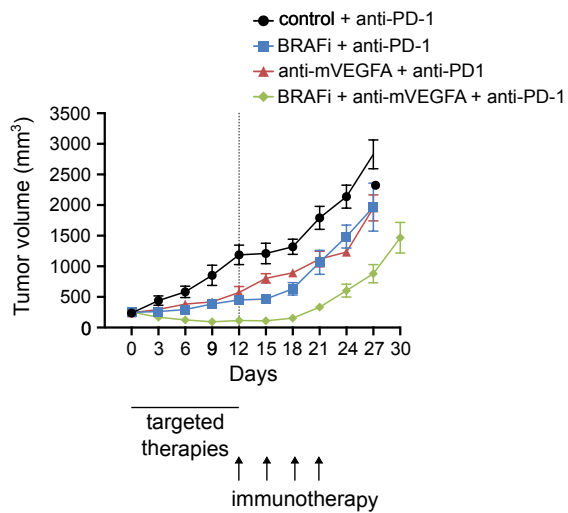

Supplement: Supplementary file 1 — Fig. S1. Syngeneic 5555 melanoma tumors are refractory to anti‐mVEGFA antibody and BRAF/VEGFA targeting does not result in synergistic antitumor activity. Fig. S2. Synergistic antitumor activity induced by BRAF/VEGFA targeting is not correlated with an augmented inhibition of tumor angiogenesis in D4M syngeneic melanoma model. Fig. S3. BRAF/VEGFA targeting delays the onset to acquired resistance to BRAFi in D4M syngeneic melanoma model. Fig. S4. Anti‐PD‐1 enhances the efficacy of BRAFi, anti‐m‐VEGFA, and their combination in D4M syngeneic melanoma model. Fig. S5. GM‐CSF neutralization and genetic knockdown demonstrates that tumor‐derived GM‐CSF regulates tumor‐clearing mechanism in D4M syngeneic melanoma model. [file MOL2-17-1474-s001.zip › Figure S4.pdf]

**A**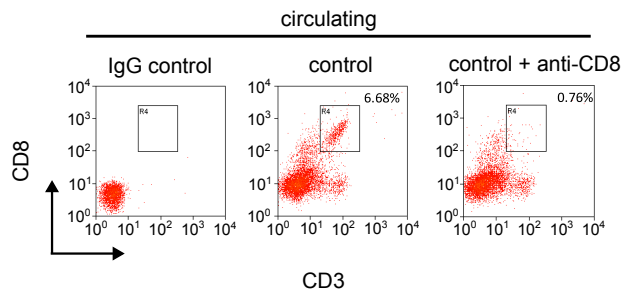**B**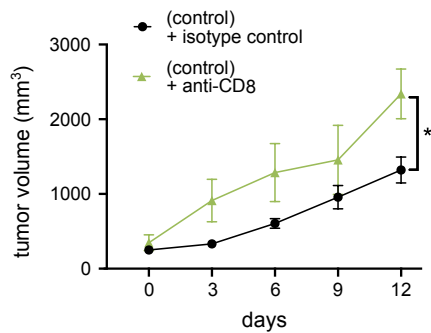**C**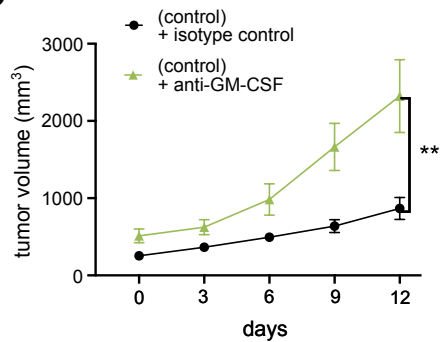**D**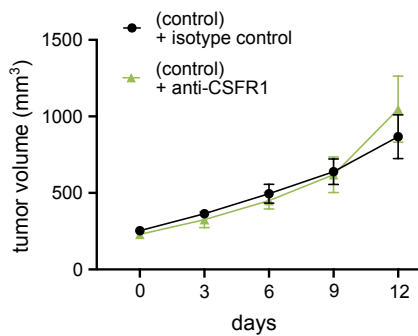**E**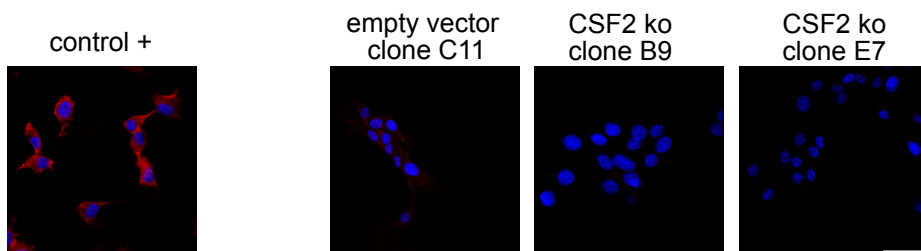**F**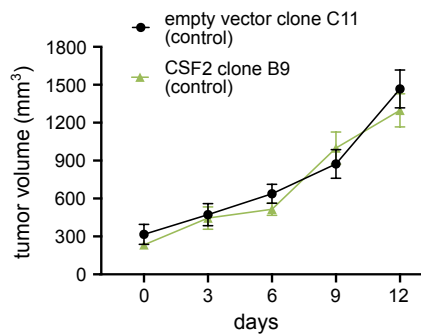**G**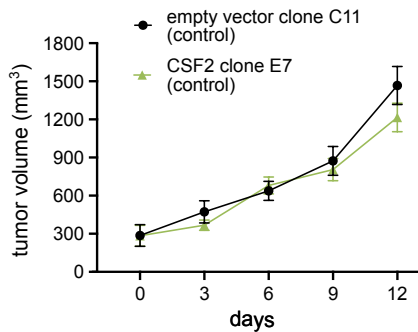

Supplement: Supplementary file 1 — Fig. S1. Syngeneic 5555 melanoma tumors are refractory to anti‐mVEGFA antibody and BRAF/VEGFA targeting does not result in synergistic antitumor activity. Fig. S2. Synergistic antitumor activity induced by BRAF/VEGFA targeting is not correlated with an augmented inhibition of tumor angiogenesis in D4M syngeneic melanoma model. Fig. S3. BRAF/VEGFA targeting delays the onset to acquired resistance to BRAFi in D4M syngeneic melanoma model. Fig. S4. Anti‐PD‐1 enhances the efficacy of BRAFi, anti‐m‐VEGFA, and their combination in D4M syngeneic melanoma model. Fig. S5. GM‐CSF neutralization and genetic knockdown demonstrates that tumor‐derived GM‐CSF regulates tumor‐clearing mechanism in D4M syngeneic melanoma model. [file MOL2-17-1474-s001.zip › Figure S5.pdf]
