# Supplementary material for: Dual VEGFA/BRAF targeting boosts PD‐1 blockade in melanoma through GM‐CSF‐mediated infiltration of M1 macrophages
Source: Mol Oncol. 2023 May 27;17(8):1474–91. doi: 10.1002/1878-0261.13450 (PMC10399721; doi:10.1002/1878-0261.13450)
Supplement: Supplementary file 2 — Table S1. Differential expression of ligand upon treatments including p value, BH adjusted, and log2ratio comparing each condition against the controls. [file MOL2-17-1474-s004.pdf]

**Table S1.** Differential expression of ligand upon treatments including p value, BH adjusted and log2ratio comparing each condition against the controls

| Gene Symbol | Gene Function | log 2 ratio BRAFi vs CTRL | p value adjusted BRAFi vs CTRL | log 2 ratio anti-hVEGF-A vs CTRL | p value adjusted anti-hVEGF-A vs CTRL | log 2 ratio BRAFi + anti-hVEGF-A vs CTRL | p value adjusted BRAFi + anti-hVEGF-A vs CTRL |
|---|---|---|---|---|---|---|---|
| *CSF2* | *Ligand* | 1.223 | 0.187 | 0.167 | 0.733 | 2.179 | 0.029 |
| *CCL2* | *Ligand* | 2.765 | 0.036 | 0.062 | 0.945 | 2.162 | 0.108 |
| *CXCL10* | *Ligand* | 1.879 | 0.036 | -0.023 | 0.945 | 2.069 | 0.029 |
| *UTS2* | *Ligand* | 2.957 | 0.221 | -0.218 | 0.945 | 2.032 | 0.405 |
| *IL8* | *Ligand* | 1.233 | 0.036 | 0.340 | 0.466 | 1.678 | 0.029 |
| *CXCL16* | *Ligand* | 1.009 | 0.122 | -0.107 | 0.896 | 1.145 | 0.392 |
| *CX3CL1* | *Ligand* | 0.860 | 0.112 | -0.444 | 0.466 | 1.073 | 0.103 |
| *LTB* | *Ligand* | 0.492 | 0.221 | 0.186 | 0.466 | 1.033 | 0.296 |
| *CCL27* | *Ligand* | 0.056 | 0.804 | 0.000 | 0.999 | 1.029 | 0.474 |
| *TGFB3* | *Ligand* | 1.246 | 0.036 | -0.389 | 0.466 | 1.006 | 0.221 |
| *TNFSF14* | *Ligand* | 0.214 | 0.767 | 0.542 | 0.577 | 1.000 | 0.029 |
| *FGF2* | *Ligand* | 0.484 | 0.197 | -0.063 | 0.896 | 0.986 | 0.254 |
| *IL18* | *Ligand* | 0.108 | 0.894 | 0.311 | 0.746 | 0.950 | 0.029 |
| *CXCL1* | *Ligand* | 1.150 | 0.011 | 0.182 | 0.733 | 0.941 | 0.405 |
| *IL15* | *Ligand* | 0.443 | 0.138 | 0.073 | 0.849 | 0.903 | 0.241 |
| *BTC* | *Ligand* | 0.921 | 0.258 | 0.108 | 0.733 | 0.806 | 0.222 |
| *PDGFD* | *Ligand* | 0.277 | 0.801 | -0.491 | 0.733 | 0.776 | 0.640 |
| *TNFSF4* | *Ligand* | 0.114 | 0.652 | -0.215 | 0.733 | 0.711 | 0.421 |
| *CXCL2* | *Ligand* | 0.637 | 0.315 | 0.250 | 0.733 | 0.705 | 0.221 |
| *BMP6* | *Ligand* | 0.425 | 0.207 | 0.235 | 0.469 | 0.672 | 0.515 |
| *BMP5* | *Ligand* | 0.665 | 0.122 | -0.027 | 0.945 | 0.650 | 0.515 |
| *IL10* | *Ligand* | -0.146 | 0.880 | 0.382 | 0.733 | 0.627 | 0.158 |
| *BDNF* | *Ligand* | 0.375 | 0.524 | -0.165 | 0.777 | 0.623 | 0.221 |
| *FGF18* | *Ligand* | 0.040 | 0.846 | 0.224 | 0.466 | 0.600 | 0.415 |
| *FBN1* | *Ligand* | 0.509 | 0.122 | -0.045 | 0.733 | 0.545 | 0.405 |
| *PTN* | *Ligand* | 0.263 | 0.721 | -0.242 | 0.777 | 0.536 | 0.608 |
| *JAG1* | *Ligand* | 0.525 | 0.215 | -0.146 | 0.733 | 0.531 | 0.405 |
| *WNT7A* | *Ligand* | 0.129 | 0.807 | 0.549 | 0.659 | 0.445 | 0.692 |
| *SPP1* | *Ligand* | 0.704 | 0.687 | 0.395 | 0.733 | 0.430 | 0.618 |
| *IL12A* | *Ligand* | 0.233 | 0.415 | 0.075 | 0.890 | 0.402 | 0.260 |
| *IL25* | *Ligand* | 0.030 | 0.904 | 0.055 | 0.896 | 0.372 | 0.640 |
| *EFNA1* | *Ligand* | -0.161 | 0.554 | -0.041 | 0.896 | 0.345 | 0.666 |
| *CXCL14* | *Ligand* | -0.395 | 0.654 | 0.924 | 0.466 | 0.330 | 0.731 |
| *VEGFA* | *Ligand* | -1.058 | 0.140 | 0.243 | 0.733 | 0.277 | 0.692 |
| *PDGFC* | *Ligand* | -0.107 | 0.804 | -0.137 | 0.733 | 0.261 | 0.598 |
| *BMP7* | *Ligand* | -0.381 | 0.652 | -0.360 | 0.733 | 0.213 | 0.866 |
| *CRLF1* | *Ligand* | -0.283 | 0.449 | -0.183 | 0.733 | 0.161 | 0.770 |
| *IL1A* | *Ligand* | 0.064 | 0.807 | 0.190 | 0.665 | 0.117 | 0.608 |
| *KITLG* | *Ligand* | 0.179 | 0.178 | 0.019 | 0.890 | 0.102 | 0.429 |
| *BMP4* | *Ligand* | -0.647 | 0.218 | -0.293 | 0.733 | 0.098 | 0.863 |
| *LIF* | *Ligand* | 0.508 | 0.576 | 0.208 | 0.733 | 0.065 | 0.948 |
| *EFNB1* | *Ligand* | 0.680 | 0.457 | 0.185 | 0.780 | 0.065 | 0.918 |
| *TNFSF11* | *Ligand* | 0.175 | 0.721 | -0.141 | 0.733 | 0.045 | 0.918 |
| *CCL20* | *Ligand* | 0.577 | 0.036 | 0.217 | 0.466 | 0.038 | 0.954 |
| *HBEGF* | *Ligand* | 0.774 | 0.267 | 0.415 | 0.466 | 0.030 | 0.948 |
| *IL1F7* | *Ligand* | 0.001 | 0.996 | -0.018 | 0.945 | 0.005 | 0.970 |
| *VEGFB* | *Ligand* | -0.389 | 0.253 | -0.134 | 0.733 | -0.011 | 0.970 |
| *SEMA3A* | *Ligand* | 0.513 | 0.256 | 0.151 | 0.746 | -0.047 | 0.948 |
| *IL6* | *Ligand* | 0.523 | 0.122 | 0.076 | 0.890 | -0.050 | 0.917 |
| *TNFSF15* | *Ligand* | 0.588 | 0.576 | 0.525 | 0.583 | -0.095 | 0.880 |
| *TNFSF12* | *Ligand* | 0.434 | 0.626 | -0.082 | 0.945 | -0.110 | 0.917 |
| *LEFTY1* | *Ligand* | 0.148 | 0.652 | -0.176 | 0.733 | -0.125 | 0.640 |
| *NRG1* | *Ligand* | 0.285 | 0.658 | 0.062 | 0.896 | -0.172 | 0.866 |
| *WNT5A* | *Ligand* | -0.132 | 0.801 | -0.180 | 0.733 | -0.191 | 0.640 |
| *GPNMB* | *Ligand* | 0.182 | 0.721 | -0.459 | 0.466 | -0.206 | 0.608 |
| *TGFA* | *Ligand* | -0.598 | 0.122 | -0.081 | 0.788 | -0.235 | 0.640 |
| *WNT3* | *Ligand* | -0.599 | 0.122 | -0.055 | 0.814 | -0.254 | 0.770 |
| *TNFSF9* | *Ligand* | -0.344 | 0.449 | 0.245 | 0.733 | -0.320 | 0.405 |
| *ADM2* | *Ligand* | -0.182 | 0.658 | -0.199 | 0.733 | -0.332 | 0.421 |
| *BMP2* | *Ligand* | -0.457 | 0.267 | 0.025 | 0.945 | -0.345 | 0.731 |
| *HMGB1L1* | *Ligand* | -0.423 | 0.652 | 0.425 | 0.466 | -0.400 | 0.640 |
| *INHA* | *Ligand* | -0.223 | 0.576 | 0.331 | 0.659 | -0.437 | 0.029 |
| *AVP* | *Ligand* | -0.179 | 0.267 | 0.005 | 0.979 | -0.442 | 0.392 |
| *EDN3* | *Ligand* | -0.270 | 0.891 | -1.063 | 0.733 | -0.457 | 0.866 |
| *EBI3* | *Ligand* | -0.104 | 0.721 | 0.185 | 0.733 | -0.518 | 0.405 |
| *GAL* | *Ligand* | -0.214 | 0.626 | 0.067 | 0.896 | -0.522 | 0.034 |
| *NRG2* | *Ligand* | 0.025 | 0.944 | -0.226 | 0.733 | -0.551 | 0.332 |
| *WNT7B* | *Ligand* | -0.379 | 0.721 | 0.175 | 0.777 | -0.631 | 0.115 |
| *DKK1* | *Ligand* | -0.831 | 0.284 | -0.837 | 0.466 | -0.667 | 0.598 |
| *WNT10B* | *Ligand* | -0.179 | 0.767 | -0.154 | 0.788 | -0.713 | 0.221 |
| *EFNA4* | *Ligand* | -0.803 | 0.309 | -0.122 | 0.788 | -0.748 | 0.360 |
| *DLL3* | *Ligand* | -0.196 | 0.652 | -0.279 | 0.733 | -0.892 | 0.405 |
| *ADM* | *Ligand* | -2.980 | 0.011 | 0.205 | 0.733 | -1.099 | 0.073 |
| *GAS6* | *Ligand* | 0.123 | 0.846 | 0.127 | 0.814 | -1.106 | 0.405 |
| *EFNB3* | *Ligand* | -0.357 | 0.449 | -0.026 | 0.950 | -1.121 | 0.405 |
| *IL11* | *Ligand* | -1.610 | 0.122 | 0.407 | 0.665 | -1.173 | 0.608 |
| *PGF* | *Ligand* | -0.211 | 0.721 | -0.125 | 0.849 | -1.248 | 0.238 |
| *CD70* | *Ligand* | -0.121 | 0.807 | -0.002 | 0.999 | -1.518 | 0.477 |
| *MDK* | *Ligand* | 0.372 | 0.567 | -0.357 | 0.733 | -1.616 | 0.583 |
| *NMB* | *Ligand* | -0.729 | 0.215 | -0.296 | 0.665 | -1.786 | 0.405 |
