# Supplementary material for: Dual VEGFA/BRAF targeting boosts PD‐1 blockade in melanoma through GM‐CSF‐mediated infiltration of M1 macrophages
Source: Mol Oncol. 2023 May 27;17(8):1474–91. doi: 10.1002/1878-0261.13450 (PMC10399721; doi:10.1002/1878-0261.13450)
Supplement: Supplementary file 3 — Table S2. Differential expression of receptor upon treatments including p value, BH adjusted, and log2ratio comparing each condition against the controls. [file MOL2-17-1474-s003.pdf]

Table S2. Differential expression of Receptor upon treatments including p value, BH adjusted and log2ratio comparing each condition against the controls

Gene Symbol	Gene Function	log 2 ratio BRAFi vs CTRL	p value adjusted BRAFi vs CTRL	log 2 ratio anti-hVEGF-A vs CTRL	p value adjusted anti-hVEGF-A vs CTRL	log 2 ratio BRAFi + anti-hVEGF-A vs CTRL	p value adjusted BRAFi + anti-hVEGF-A vs CTRL
Acvrl1	*Receptor*	0.202	0.776	0.733	0.244	-0.017	0.993
Fzd2	*Receptor*	0.305	0.380	0.124	0.900	-0.006	0.993
Il1rap	*Receptor*	-0.008	0.964	-0.114	0.852	-0.001	0.993
Tlr2	*Receptor*	1.007	0.017	0.235	0.459	-0.113	0.970
Mertk	*Receptor*	0.434	0.420	-0.032	0.980	-0.097	0.968
Tnfrsf11a	*Receptor*	0.109	0.865	-0.388	0.764	-0.131	0.935
Sdc4	*Receptor*	0.320	0.699	0.409	0.811	0.105	0.921
Avpr1a	*Receptor*	0.303	0.608	-0.094	0.900	0.194	0.904
Ephb1	*Receptor*	0.051	0.921	-0.570	0.417	-0.209	0.904
Erbb2	*Receptor*	0.107	0.776	0.083	0.943	0.071	0.904
Fzd7	*Receptor*	-0.070	0.837	0.129	0.900	-0.067	0.904
Il10ra	*Receptor*	0.974	0.043	0.417	0.331	0.177	0.904
Notch1	*Receptor*	0.122	0.699	-0.571	0.244	0.062	0.904
Cd44	*Receptor*	-0.423	0.654	0.118	0.900	-0.077	0.903
Itgb6	*Receptor*	0.181	0.776	-0.431	0.396	0.247	0.874
Lrp6	*Receptor*	-0.373	0.635	-0.715	0.361	-0.208	0.873
Kit	*Receptor*	-0.119	0.732	-0.626	0.331	-0.231	0.852
Ptpn6	*Receptor*	0.391	0.260	0.367	0.353	-0.335	0.834
Cxcr4	*Receptor*	-0.120	0.687	0.064	0.943	-0.299	0.799
Tnfrsf11b	*Receptor*	0.099	0.837	-0.007	0.988	0.326	0.799
Il10rb	*Receptor*	0.315	0.654	0.425	0.732	0.379	0.769
Brs3	*Receptor*	-0.093	0.776	-0.171	0.852	-0.185	0.754
Ccr5	*Receptor*	-0.706	0.588	0.214	0.900	-0.865	0.754
Ednra	*Receptor*	0.662	0.339	-0.017	0.988	0.530	0.754
Egfr	*Receptor*	1.068	0.339	0.116	0.949	0.788	0.754
Fgfrl1	*Receptor*	0.507	0.310	0.243	0.735	0.227	0.754
Nrp1	*Receptor*	0.467	0.252	-0.395	0.479	0.168	0.754
Pdgfra	*Receptor*	1.045	0.339	0.378	0.853	0.702	0.754
Tgfbr3	*Receptor*	0.703	0.515	0.132	0.943	-0.483	0.754
Tlr4	*Receptor*	0.200	0.687	-0.130	0.900	-0.460	0.754
Tnfrsf4	*Receptor*	-0.099	0.762	-0.159	0.900	0.283	0.754
Fzd5	*Receptor*	0.547	0.260	-0.142	0.900	0.493	0.735
Csf2ra	*Receptor*	0.362	0.635	0.107	0.943	0.380	0.710
Cx3cr1	*Receptor*	-0.146	0.742	-0.517	0.361	-1.143	0.710
Il11ra1	*Receptor*	0.843	0.252	0.125	0.900	0.764	0.710
Acvr1b	*Receptor*	0.352	0.553	0.291	0.717	0.290	0.706
Bmpr1a	*Receptor*	1.061	0.170	-0.061	0.963	0.898	0.706
Fzd6	*Receptor*	0.293	0.403	0.111	0.811	0.682	0.706
Rgma	*Receptor*	0.542	0.339	0.060	0.900	0.815	0.706
Il17rc	*Receptor*	0.709	0.063	0.302	0.735	1.140	0.703
Mela	*Receptor*	1.675	0.371	1.302	0.582	1.245	0.702
Epha3	*Receptor*	0.289	0.635	-0.007	0.988	-0.180	0.702
Ltbr	*Receptor*	0.453	0.380	0.121	0.900	-0.305	0.702
Sostdc1	*Receptor*	-0.083	0.339	0.002	0.988	1.077	0.702
Fzd1	*Receptor*	0.630	0.077	0.121	0.732	0.873	0.695
Sphk2	*Receptor*	0.253	0.715	-0.296	0.764	-0.262	0.638
Il15ra	*Receptor*	1.331	0.043	0.443	0.459	0.458	0.628
Il1r2	*Receptor*	0.512	0.403	0.670	0.361	0.933	0.628
Itgb1	*Receptor*	0.083	0.806	0.026	0.988	-0.488	0.628
Epha2	*Receptor*	-0.105	0.635	0.123	0.732	-0.131	0.555
Il6ra	*Receptor*	0.777	0.260	0.754	0.331	0.671	0.549
Tnfrsf9	*Receptor*	-0.123	0.654	0.334	0.703	-0.330	0.549
Galr2	*Receptor*	0.997	0.043	-0.082	0.459	0.614	0.545
Il27ra	*Receptor*	0.720	0.130	0.129	0.900	0.985	0.545
Il6st	*Receptor*	0.499	0.252	-0.025	0.900	0.265	0.476
Tgfbr1	*Receptor*	0.656	0.099	-0.134	0.852	0.509	0.408
Tgfbr2	*Receptor*	1.034	0.043	0.148	0.852	0.407	0.408
Itgb5	*Receptor*	0.483	0.260	-0.015	0.988	0.459	0.381
Cxcr3	*Receptor*	0.323	0.260	0.103	0.417	0.993	0.375
Darc	*Receptor*	1.116	0.252	0.727	0.459	1.195	0.331
Il12rb1	*Receptor*	0.083	0.528	0.127	0.246	0.175	0.331
Sort1	*Receptor*	0.908	0.339	-0.052	0.952	1.805	0.331
Cd27	*Receptor*	0.615	0.260	0.516	0.246	1.488	0.269
Il18r1	*Receptor*	0.742	0.100	0.073	0.749	1.770	0.269
Ly96	*Receptor*	0.824	0.260	0.829	0.246	1.249	0.269
Ptprb	*Receptor*	-0.141	0.654	-0.384	0.361	-0.749	0.269
Ephb4	*Receptor*	-0.399	0.283	-0.341	0.396	-1.010	0.096
Tlr1	*Receptor*	0.399	0.339	-0.234	0.852	0.691	0.085
Pdgfrb	*Receptor*	0.858	0.298	0.006	0.988	-0.747	0.080
Ednrb	*Receptor*	-0.477	0.100	-1.023	0.212	-1.136	0.049
Ramp3	*Receptor*	-1.539	0.037	-0.562	0.459	-1.397	0.049
Flt1	*Receptor*	-0.916	0.007	-0.900	0.005	-1.220	0.044
