# Supplementary material for: Dual VEGFA/BRAF targeting boosts PD‐1 blockade in melanoma through GM‐CSF‐mediated infiltration of M1 macrophages
Source: Mol Oncol. 2023 May 27;17(8):1474–91. doi: 10.1002/1878-0261.13450 (PMC10399721; doi:10.1002/1878-0261.13450)
Supplement: Supplementary file 4 — Table S3. Log2 Fold Change Real‐Time PCR analysis of macrophages polarization. [file MOL2-17-1474-s002.pdf]

Table S3. Log2 Fold Change Realt Time PCR ananlysis of macrophages polarization

Gene	Control (n=4)	BRAFi (n=4)	anti-mVEGF-A (n=4)	BRAFi + anti-mVEGF-A (n=4)
Cxcl10	0.005	2.985	2.525	3.540
Cd40	0.005	3.960	3.275	4.460
Cd86	0.000	2.055	1.535	2.480
Cxcl9	0.000	3.875	3.960	5.170
Ccl5	0.000	5.505	3.400	6.140
Tnfa	0.000	2.995	0.695	2.345
Nos2	-0.435	1.205	4.985	3.240
Arg1	0.000	-2.935	2.340	-0.585
